# Sperm-Specific CatSper is Not Conserved in All Vertebrates and May Not be the Only Progesterone-Responsive Ion Channel Present in Sperm

**DOI:** 10.1007/s00232-024-00316-1

**Published:** 2024-07-06

**Authors:** Nishant Kumar Dubey, Vikash Kumar, Chandan Goswami

**Affiliations:** 1 School of Biological Sciences, National Institute of Science Education and Research Bhubaneswar, P.O. Jatni, Khurda, 752050 Odisha India; 2 Training School Complex, Homi Bhabha National Institute, Anushakti Nagar, Mumbai, 400094 India

**Keywords:** Molecular evolution, Ca^2+^-channel, Lipid-water-interface, Molecular pattern, TRPV4

## Abstract

Progesterone (P4) acts as a key conserved signalling molecule in vertebrate reproduction. P4 is especially important for mature sperm physiology and subsequent reproductive success. “CatSpermasome”, a multi-unit molecular complex, has been suggested to be the main if not the only P4-responsive atypical Ca^2+^-ion channel present in mature sperm. Altogether, here we analyse the protein sequences of CatSper1-4 from more than 500 vertebrates ranging from early fishes to humans. CatSper1 becomes longer in mammals due to sequence gain mainly at the N-terminus. Overall the conservation of full-length CatSper1-4 as well as the individual TM regions remain low. The lipid-water-interface residues (i.e. a 5 amino acid stretch sequence present on both sides of each TM region) also remain highly diverged. No specific patterns of amino acid distributions were observed. The total frequency of positively charged, negatively charged or their ratios do not follow in any specific pattern. Similarly, the frequency of total hydrophobic, total hydrophilic residues or even their ratios remain random and do not follow any specific pattern. We noted that the CatSper1-4 genes are missing in amphibians and the CatSper1 gene is missing in birds. The high variability of CatSper1-4 and gene-loss in certain clades indicate that the “CatSpermasome” is not the only P4-responsive ion channel. Data indicate that the molecular evolution of CatSper is mostly guided by diverse hydrophobic ligands rather than only P4. The comparative data also suggest possibilities of other Ca^2+^-channel/s in vertebrate sperm that can also respond to P4.

**Graphical Abstract:**

In birds, only CatSper1 is absent. Similarly, in amphibians, all the CatSper units are absent, suggesting the presence of other proteins that can act as P4-responsive Ca^2+^-ion channels there. TRPV4 is present in all these vertebrate groups and multiple copies of the TRPV4 gene are present in amphibians
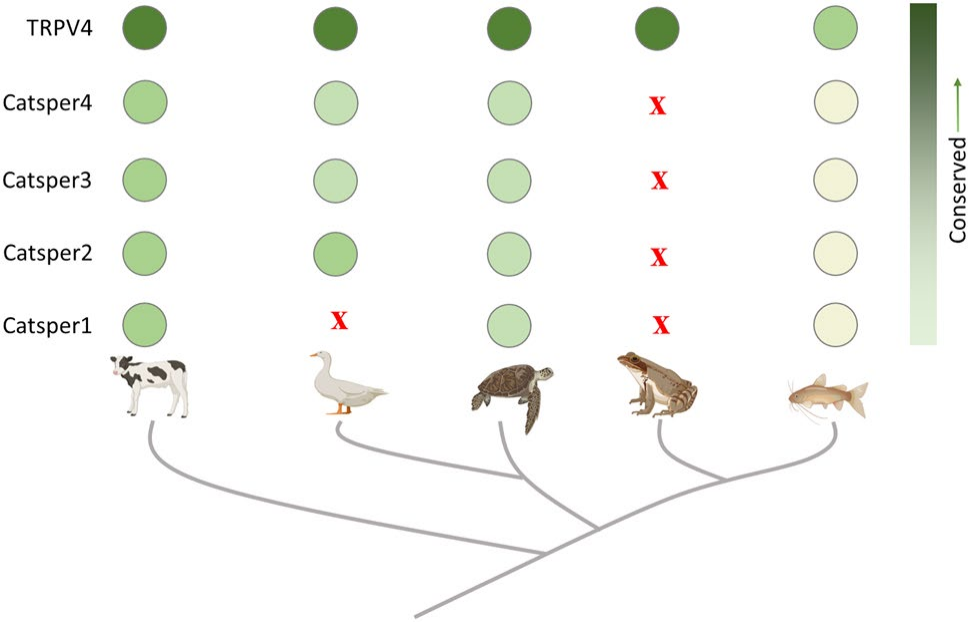

**Supplementary Information:**

The online version contains supplementary material available at 10.1007/s00232-024-00316-1.

## Introduction

Reproductive success is a key event that drives evolution. In that context, sexual reproductions across the species undergo a lot of challenges, and sperm cells in particular need to adapt to diverse environmental challenges. To accommodate different adaptations, sperm cells are equipped with different sets of molecular tools and mechanisms. Mature sperm cells undergo a series of physiological changes step-by-step, such as Ca^2+^-influx, protein phosphorylation, hyper-activation, and acrosomal reaction which all are required for successful fertilization (Gervasi and Visconti. 2016). Therefore, functions like reproductive success, speciation, and species evolution, are often guided by the molecular evolution of key molecules present in the sperm cells and their precise structure–function relationships.

Progesterone (P4) acts as an important endogenous sex steroid hormone and it regulates a plethora of physiological responses (Wiltbank et al. 2014). Among all, the involvement of P4 in reproductive functions are important and critical for reproductive success (Nagy et al. 2021). P4 stimulates mature sperm cells from different species and causes hyperactivation, a Ca^2+^-driven physiological step important for successful reproduction (Suzuki and Fujinoki. 2023). Thus, P4-mediated signaling events and the key molecules involved in P4-sensing are expected to be critical and play an important role in evolutionary success.

It was reported that the CatSper ion channel is the Ca^2+^ ion channel which is responsive to P4 and is important for P4-mediated cell signaling (Strünker et al. 2011). Notably, CatSper is present in sperm cells across different vertebrates and also in invertebrates (Cai and Clapham. 2008). Animals that are genetically depleted with CatSper (knock out) have complete male sterility. Any mutation in the CatSper ion channel gene will hinder fertilization (Brown et al. 2019; Sun et al. 2017). The pore-forming subunits are made of CatSper alpha (α) 1, 2, 3, and 4. All these have 6 transmembrane regions. In addition, there are six auxiliary subunits namely beta (β), gamma (γ), delta (δ), epsilon (ε), zeta (ζ), and EFCAB9 which all are required for sperm function/s (Lin et al. 2021). All these sub-units together form a functional complex, which is known as “CatSpermasome” and CatSper1-4 are the major components of the core ion channel. Though the initial literature suggested CatSper is the main ion channel responsible for P4-mediated Ca^2+^-influx and hyperactivation in sperm, the later findings suggest many other alternatives. The involvement of CatSper as the sole ion channel responsible for the P4-sensing ability present in mature sperm remains debatable for many reasons (discussed later).

The transmembrane region and nearby regions, such as loops of different ion channels interact with different hydrophobic ligands and lipids present in the membrane. For channel activation, the TM regions as well as loop regions of ion channels undergo a series of conformational changes and their relative spatio-temporal positions concerning lipid bilayer alters. Therefore, specific amino acids present in the TM and loop regions determine the channel function and regulations, mainly due to their side chains that impose flexibility-rigidity towards conformational changes required for proper channel function. The side chains of amino acids also determine their orientation and stability in the lipid membrane, especially at the lipid-water-interface (LWI) region. The LWI region is defined as the narrow layer (of ~ 6–10 Å on the Z-axis) located on both sides of the membrane where water concentration is very low but not completely absent. Thus, the LWI region offers a unique microenvironment that determines the enrichment of several ligands and subsequently regulation of transmembrane proteins such as ion channels, pumps, and receptors. Therefore, systemic studies of molecular selection or exclusion of specific amino acids located in the LWI region provide useful information about the molecular evolution driven by the regulation and/or function of specific ion channels.

Due to the advancement of genome sequencing, many full-length sequences are available from diverse organisms. Comparative analysis of sequence length, and amino acids present in the LWI region helps to identify the conservation, pattern formation, and common factors involved and thus allows to predict the function as well as regulation of the protein across different taxa. In this work, we analysed the protein sequence of CatSper1-4, the main 4 subunits of CatSpermasome. We critically evaluated the sequence length and compared different structural and functional units to understand the molecular evolution of CatSper1-4. We also analysed the conservation and amino acid compositions of lipid-water-interface regions of CatSper1-4 for the possible presence of specific patterns. Our data suggest that the LWI regions of CatSper1-4 are highly variable and do not have any specific pattern, suggesting that CatSpermasome may not be the sole molecular complex responsible for P4-signalling in sperm.

## Materials and Method

### Sequence Retrieval and Alignment

The human CatSper1 sequences were retrieved from NCBI (NP_444282.3) and multiple BLAST runs were performed to retrieve the sequences from all other vertebrates (Supplementary Table 1). The amino acid sequence of a total of 98 species was collected from all vertebrates which included 75 mammals, 10 reptiles, and 13 fishes. Similarly, the human CatSper2 sequence from NCBI (NP_001269238.1) was retrieved and a BLAST search gave 137 species including 84 mammals, 6 birds, 27 reptiles, and 20 fishes. For the CatSper3 sequence human (NP_821138.1) was used to run a BLAST and 150 species were collected including 96 mammals, 9 birds, 25 reptiles, and 20 fishes. For the CatSper4 sequences, human (XP_011539734.1) was used to run a BLAST, and 137 species were selected with 90 mammals, 3 birds, 23 reptiles, and 21 fishes. All samples were aligned using MUSCLE from MEGA 11.0 software. Taking human as a reference, the transmembrane region (TM) was selected for all 4 CatSper proteins, and from the aligned files the LWI regions and TM region were selected for all the analysis.

### Selection of Transmembrane Region

The different prediction models namely TMHMM, DeepTMHMM and TMSEG were used to predict the transmembrane region of the CatSper1, 2, 3, and 4 of human protein (Bernhofer et al. 2016; Hallgren et al. 2022; Krogh et al. 2001).

### Calculation of Amino Acid Frequencies in the Lipid-Water-Interface Region

Human samples from UniProt ID-Q8NEC5 for CatSper1, ID-Q96P56 for CatSper2, ID-Q86XQ3 for CatSper3, and ID-Q7RTX7 for CatSper4, respectively, were taken as a reference for the transmembrane region and visualized using *wlab.ethz.ch/protter*. All 6 transmembrane regions were identified, and 5 amino acid-long stretches in the C-terminal side of the transmembrane and the N-terminal side of the transmembrane regions are considered as the Lipid-Water Interface (LWI) residues.

The LWI regions were selected and amino acid frequencies were calculated using MEGA 11.0 software and plotted in GraphPad Prism 9.0. The amino acids were arranged according to ∆G values of the side chain of the amino acids as suggested before (Wimley and White. 1996). Snorkeling propensities of each amino acid were also considered as suggested before (Chamberlain et al. 2004)*.* The Kyte-Doolite scale was used for the calculation of hydrophobicity (Kyte and Doolittle. 1982). In certain calculations, frequencies of hydrophobic amino acids (namely Tyrosine, Isoleucine, Leucine, Methionine, Phenylalanine, Cysteine, and Tryptophan) were combined and the total frequency was plotted. Similarly, frequencies of hydrophilic amino acids (namely Alanine, Glycine, Histidine, Proline, Serine, Threonine, Valine, Lysine, Arginine, Glutamic acid, Aspartic acid, Glutamine, and Asparagine) were combined and the total value was plotted. In some analysis, frequencies of positive amino acids namely Arginine, Histidine, and Lysine, and frequencies of negative amino acids Aspartic acid and Glutamic acid were combined. These values for outer LWI, inner LWI, and total LWI regions were calculated and plotted.

### Amino Acid Enrichment and Depletion Generator in the LWI Region

The enrichment of amino acids at the LWI region across the different taxon was generated. It identifies the most represented amino acid in the aligned sequences at that region. The amino acids depletion that is under-represented of amino acids in the same region was identified and the logo was generated using Kullback–Leibler (Thomsen and Nielsen 2012). The positive Y-axis represents the enrichment of amino acids whereas the negative Y-axis represents the depletion of amino acids. Logos were generated using the Seq2logo (services.healthtech.dtu.dk/services/Seq2Logo-2.0/).

### Boxplot Generation

The conservation of different regions was calculated by a pairwise distance matrix from Mega 11.0 software. First, the aligned files were selected and their variance was estimated using Bootstrap with 1000 replications. The substitution model was p-distance and with uniform rates among sites. In certain cases, the sequence variation results in improper alignments (gaps/missing data treatment), and such sequences were subject to complete deletion from the data set. The distance matrix values were plotted as a box plot using GraphPad prism.9. In this analysis, the score remains within 0–1 where 0 or 1 value indicates highly conserved and highly diverged protein (or protein segment), respectively.

### Sperm Collection from Bull

Frozen bull semen straws were obtained from FSB, Khapuria, Cuttack, India. These straws were thawed in a 37 °C water bath for 55 s and immediately transferred into pre-warmed modified Human Tubal Fluid (mHTF) buffer which contains NaCl (101 mM), KCl (4.69 mM), MgSO_4_.7H_2_O (0.2 mM), KH_2_PO_4_ (0.4 mM), CaCl_2_.2H_2_O (5.14 mM), Glucose (2.78 mM), NaHCO_3_ (25 mM), Na-Lactate (18.36 mM), Na-Pyruvate (0.34 mM), pH 6.8 and maintained at 37ºC and left undisturbed for 30 min away from light to allow the sperm to swim out (Kito and Ohta 2005). The Swim-out samples were collected and motility analysis were performed.

### Motility Assay of Bull Sperm

The Swim-out bull sperm was collected and kept in different conditions by incubating them with or without the following drugs: Progesterone (P4, Sigma, 100 nM), GSK1016790A (a potent TRPV4 ion channel activator, Tocris, 100 nM), RN1734 (a potent TRPV4 ion channel antagonist, Tocris, 10 μM), NNC550396 (a potent Catsper ion channel antagonist, Sigma, 5 μM) at 37 °C. In some conditions, the sperms were preincubated with these drugs for 1 min in the order as and when mentioned in the figure. The condition in which no drug was added was taken as a control. After incubation, the samples were put on pre-warmed microscopic slides and motility was measured using the Computer Assisted Semen Analysis (CASA) system. A minimum of 200 sperms were examined in each condition.

### Statistical Analysis

Pairwise distances among different groups were plotted using GraphPad Prism 9.0. The appearance was selected as the box and whiskers with the whiskers set at minimum and maximum. All the tests were performed in the SciPy Python library. The values were tested for significance using the Chi-square test. The Chi-square statistics mentioned in figure legends. All the groups are independent and with P < 0.001.

## Result

### The Protein Sequence Length of CatSper1-4 Remains Highly Variable

**Fig. 1:**
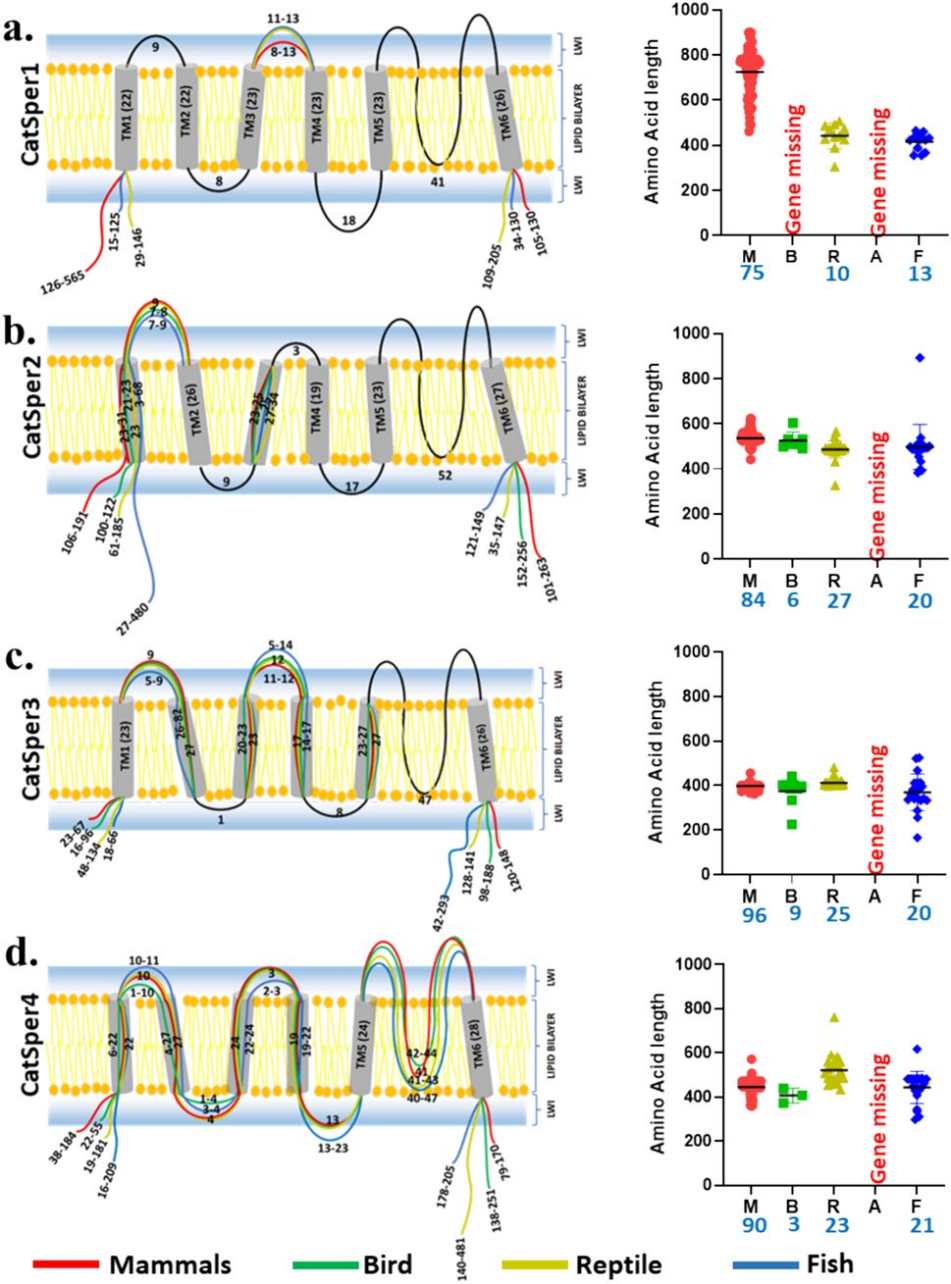
Polypeptide lengths of CatSper1-4 subunits remain variable in different phyla. a–d The polypeptide lengths of CatSper1–4 in Mammals, Birds, Reptiles, and Fishes were shown schematically as per their length and presence or absence in different genomes. The N-terminal region shows much variable sequence length for CatSper1. The Lipid-Water-Interface region is 5–10 Å in thickness on both sides of the lipid bilayer. Reptiles have longer sequence lengths in the C-terminal for CatSper4. In each case, the sequence length of different species is plotted according to the phyla on the right side. The number of species analyzed in each group is indicated in the graphs

We analysed the total length of CatSper1-4 units from multiple species and we noted that the length of CatSper1 remains highly variable whereas the total lengths of CatSper2-4 are not that variable (Fig. 1a–d). The overall lengths of CatSper1-4 were compared across the different phyla of vertebrates (Fig. 1a–d). The data suggest that the majority of CatSper1 from mammals are longer and have gained sequences mainly at the N-terminus. This accords well with the fact that the N-terminus of CatSper1 is significantly longer (300–400 amino acids in different species) in mammals and often shows the incorporation of multiple Histidine residues there. For example, within the entire N-terminal region of CatSper1, the Histidine residue constitutes ~ 16–18% in the case of most of the mammals including humans. We noted minor changes in the length of the TM regions and loop regions of CatSper1-4. We also noted length-wise conservation in certain segments.

### The TM Regions and Lipid-Water-Interface Segments are Less Conserved in CatSper1-4

**Fig. 2:**
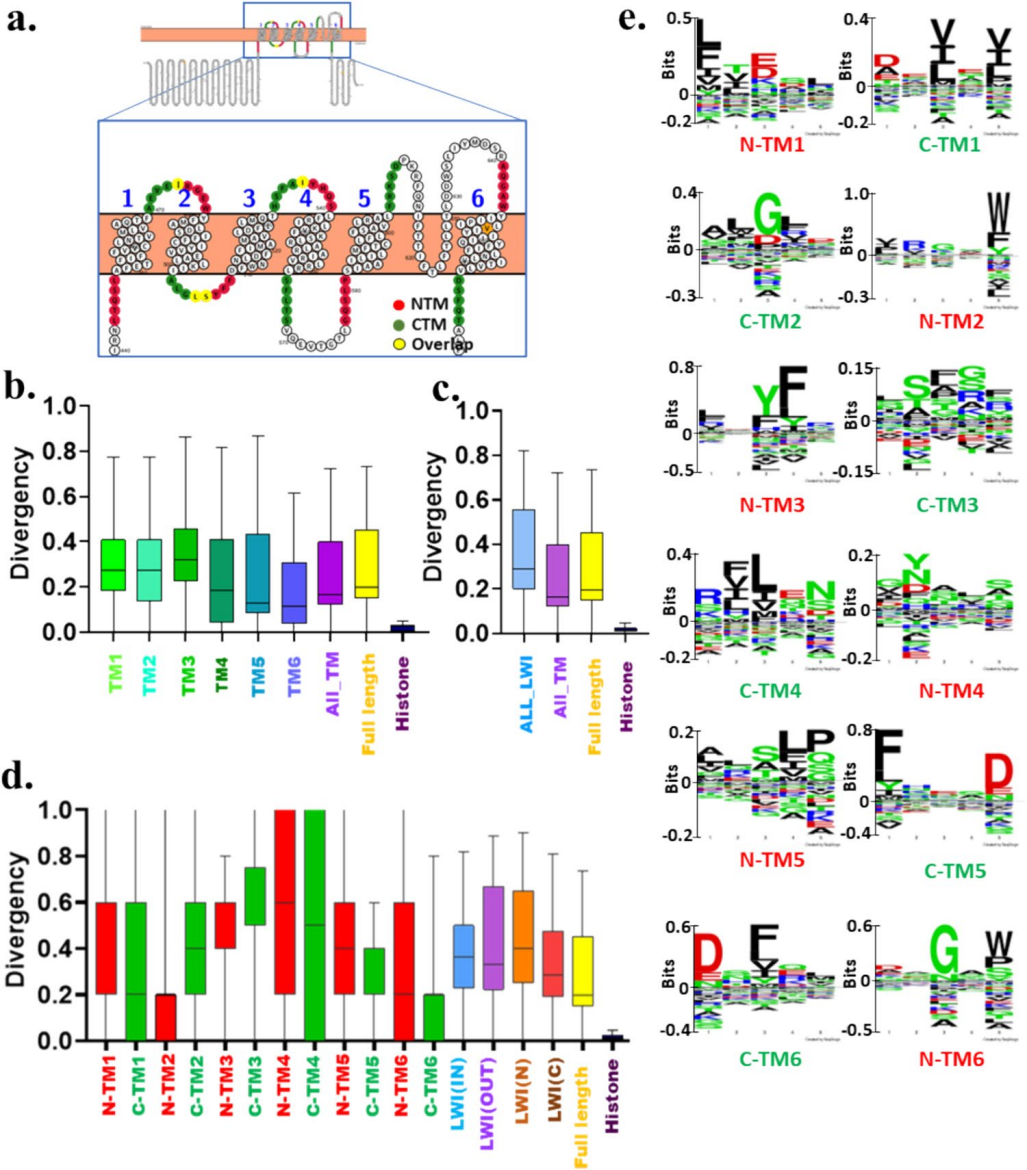
Neither full-length CatSper1 nor its fragments are conserved in vertebrates. **a** Shown is the schematic position of 5 amino acid stretches on both sides of each transmembrane that serves as the lipid-water-interface (LWI) residues. The human CatSper1 (UniProt ID-Q8NEC5) sequence was visualized on the protter website and shown as the reference. The N-terminal and C-terminal sides of the TM regions representing LWI residues are depicted in red and green, respectively. Due to the short sequence, the C-terminal of TM1 (C-TM1) and N-terminal of TM2 (N-TM2) are overlapped (marked in yellow) and such overlapping residues are counted for both C-TM1 as well as for N-TM2. This is also the case for C-TM2—N-TM3 and C-TM3—N-TM4 regions. **b** Conservation analysis of different regions of CatSper1 across vertebrate evolution is shown. None of the TM regions show high levels of conservation. **c** The LWI regions are less conserved than the TM or full-length CatSper1, suggesting overall more selection pressure on the TM residues than the LWI segments. **d** All the LWI regions are diverse. LWI (IN) is more conserved than LWI (OUT). **e** Conservation of individual residues in the LWI region is analysed and aromatic amino acids such as Tryptophan, Tyrosine, and Phenylalanine are found to be highly enriched. The independence of the group was estimated using the Chi-square test. χ^2^ = 772,498.138, 56,239.551 and 772,370.550 for b, c and d, respectively

Next, we analysed the conservation of TM segments and associated LWI regions of CatSper1. All the six TM regions of CatSper1 are less conserved, though the TM5 and TM6 are better conserved among all the TM regions (Fig. 2a–b). Notably, the total TM regions are more conserved than the full-length CatSper1 (Fig. 2c). In the same comparatives, the total lipid-water-interface residues are less conserved than the TM regions or even full-length CatSper1, suggesting much less selection pressure in the LWI regions (Fig. 2c). Analysis of individual twelve LWI residues also suggests that none of these LWI regions are conserved (Fig. 2d). Yet N-TM2 and C-TM6 are better conserved among all. Notably, in a comparative manner, inner LWI regions are more conserved than the outer LWI regions (Fig. 2d). Further Seq2Logo analysis also indicates that the LWI regions are not conserved. However, position-specific enrichment of aromatic amino acids (such as Tyrosine, Tryptophan, and Phenylalanine residues) is notable (Fig. 2e).

**Fig. 3:**
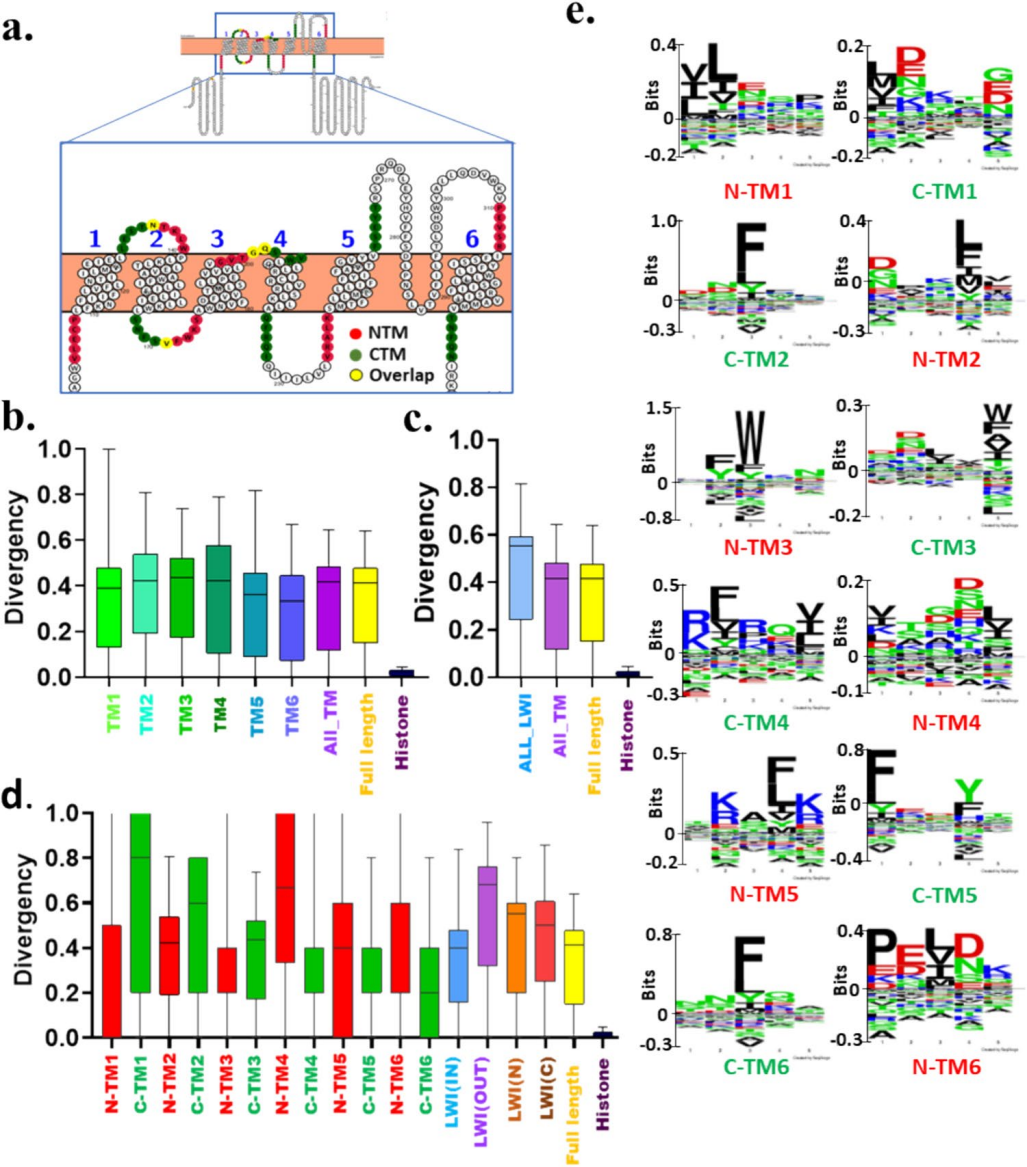
Neither full-length CatSper2 nor its fragments are conserved in vertebrates. **a** Shown is the schematic position of 5 amino acid stretches on both sides of each transmembrane that serves as the lipid-water-interface (LWI) residues. The human CatSper2 (UniProt ID-Q96P56) sequence was visualized on the protter website and shown as the reference. The N-terminal and C-terminal sides of the TM regions representing LWI residues are depicted in red and green, respectively. Note that due to the short sequence the C-terminal of TM3 (C-TM3) and N-terminal of TM4 (N-TM4) are overlapped (shown in yellow) and are counted for both C-TM3 as well as for N-TM4. This is also the case for C-TM2 and N-TM3. This is also the case for C-TM1-N-TM2 regions. **b** Conservation analysis of different regions of CatSper2 during vertebrate evolution is shown. None of the TM regions show high levels of conservation. **c** The LWI regions are less conserved than the TM or full-length sequence of CatSper2, suggesting overall more selection pressure on the TM residues than the LWI segments. **d** All the LWI regions are diverse. LWI (IN) is more conserved than LWI (OUT). **e** Conservation of individual residues in the LWI region is analysed and aromatic amino acids, such as Tryptophan, Tyrosine, and Phenylalanine are found to be highly enriched. The independence of the group was estimated using the Chi-square test. χ^2^ = 563,141.757, 100,343.304, and 1,753,373.965 for **b**, **c** and **d,** respectively

Similarly, we analysed the conservation of CatSper2-4. Neither any of the six TM regions nor any of the twelve LWI regions are conserved in the case of CatSper2 (Fig. 3a–d). The overall LWI regions are less conserved than the associated TM regions, suggesting that for CatSper2, the relative selection pressure is low for LWI residues (Fig. 3c). Though none of the individual twelve LWI are conserved, we noted that the inner LWI region is more conserved than the outer LWI (Fig. 3d). Like the CatSper1, the Seq2Logo of CatSper2 highlights the enrichment of aromatic amino acids like Phenylalanine and Tryptophan. (Fig. 3e).

**Fig. 4:**
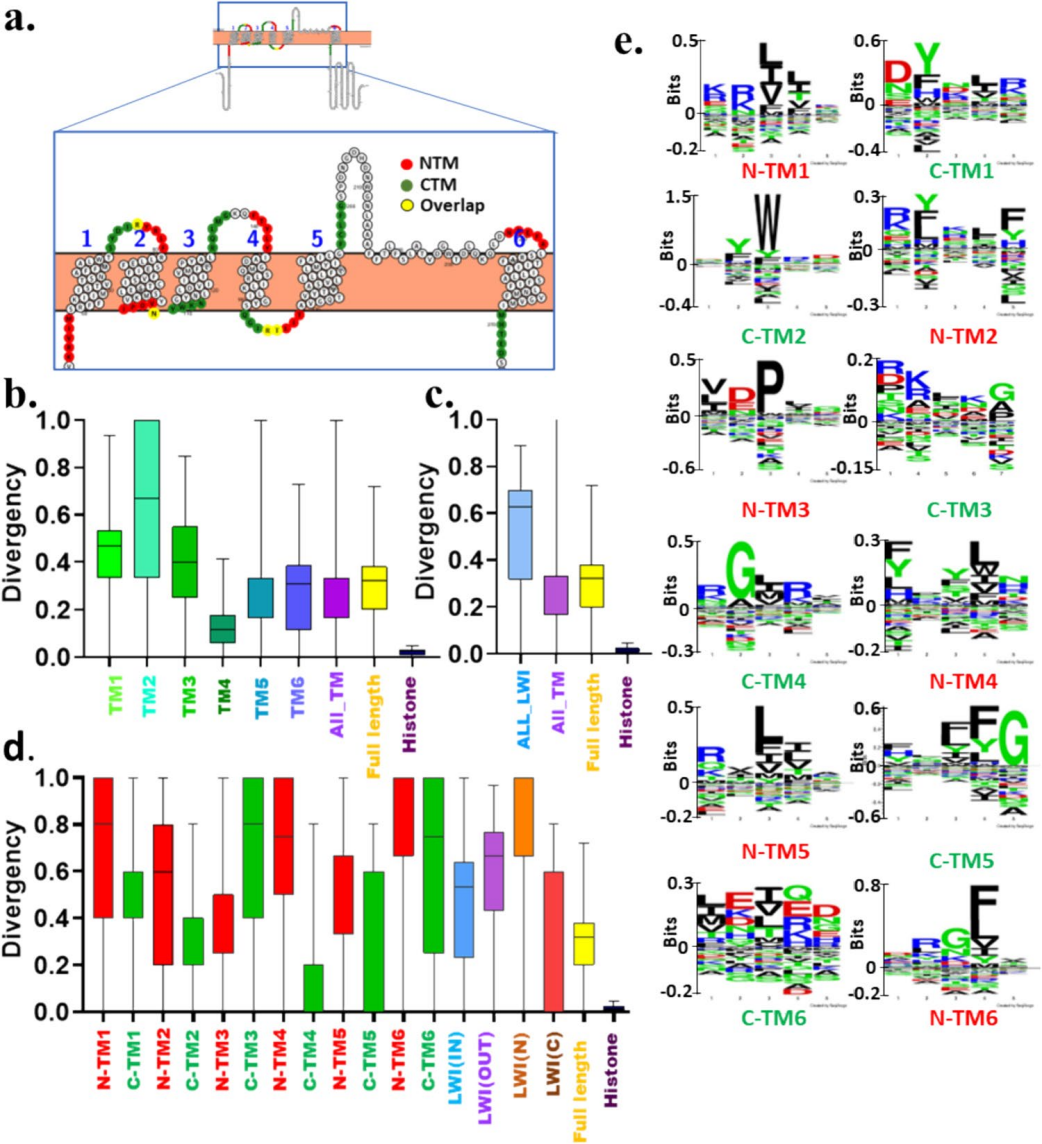
Neither full-length CatSper3 nor its fragments are conserved in vertebrates. **a** Shown is the schematic position of 5 amino acid stretches on both sides of each transmembrane that serves as the lipid-water-interface (LWI) residues. The human CatSper3 (UniProt ID- Q86XQ3) sequence was visualized on the protter website. **b** The N-terminal and C-terminal sides of the TM regions representing LWI are depicted in red and green, respectively. Note that due to the short sequence, the C-terminal of transmembrane (C-TM1) and N-terminal of transmembrane 3 (N-TM2) are overlapped (shown in yellow) and are counted for both C-TM1 as well as for N-TM2. This is also the case for (C-TM2) and (N-TM3). **c** Box plot analysis of different regions of CatSper3 across vertebrate evolution is shown. **d** The TM regions are more conserved than the LWI or full-length sequence of CatSper3, suggesting overall more selection pressure on the TM residues than the LWI segments. **e** Conservation of individual residues in the LWI region is analysed and aromatic amino acids, such as Tryptophan, Tyrosine, and Phenylalanine are found to be highly enriched. The independence of the group was estimated using the Chi-square test. χ^2^ = 448,996.458, 101,097.642 and 909,394.412 for b, c and d, respectively

Neither any of the six TM regions nor any of the twelve LWI regions are conserved in the case of CatSper3 (Fig. 4a–d). The overall LWI regions are less conserved than the associated TM regions also (Fig. 4c). However, among all, TM4 is better conserved (Fig. 4b). Also, C-TM4 is better conserved among all the twelve LWI regions (Fig. 4d). Notably, the LWI regions are more variable than the associated TM regions, suggesting that for CatSper3, the relative selection pressure is low for LWI residues. Incidentally, the inner LWI is more conserved than the outer LWI in CatSper3 (Fig. 4d). The Seq2Logo analysis shows the enrichment of Phenylalanine in N-TM6 (Fig. 4e).

**Fig. 5:**
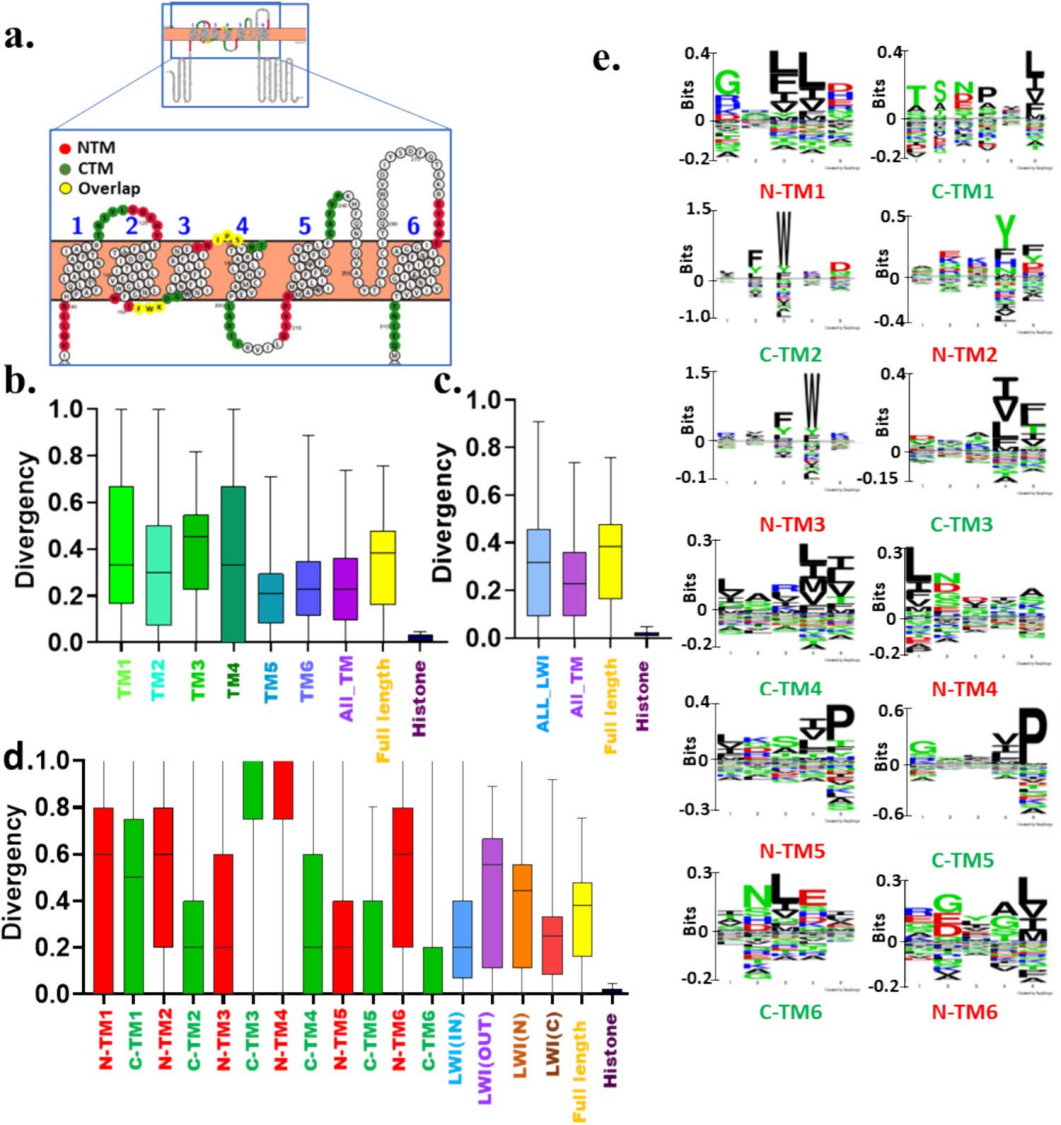
Neither full-length CatSper4 nor its fragments are conserved in vertebrates. **a** Shown is the schematic position of 5 amino acid stretches on both sides of each transmembrane that serves as the lipid-water-interface (LWI) residues. The human CatSper4 (UniProt ID-Q7RTX7) sequence was visualized on the protter website. **b** The N-terminal and C-terminal sides of the TM regions representing LWI are depicted in red and green, respectively. Note that due to the short sequence, the C-terminal of transmembrane (C-TM2) and N-terminal of transmembrane 3 (N-TM3) are overlapped and are counted for both C-TM2 as well as for N-TM3. This is also the case for C-TM3 and N-TM4. **c** Conservation analysis of different regions of CatSper4 during vertebrate evolution is shown. **d** The TM regions are more conserved than the LWI or full-length sequence of CatSper4, suggesting overall more selection pressure on the TM residues than the LWI segments. **e** Conservation of individual residues in the LWI region is analysed and aromatic amino acids i.e. Tryptophan, are found to be highly enriched. The independence of the group was estimated using the Chi-square test. χ^2^ = 465,244.406, 94,188.9201042255.276 for b, c, and d, respectively

Neither any of the six TM regions nor any of the twelve LWI regions are conserved in the case of CatSper4, except N-TM6 (Fig. 5a-d). The overall LWI regions are less conserved than the associated TM regions also (Fig. 5c). However, among all, TM5 is better conserved (Fig. 5b). The N-TM6 is better conserved among all the twelve LWI regions (Fig. 5d). Notably, the LWI regions are more variable than the associated TM regions, suggesting that for CatSper4, the relative selection pressure is low for LWI residues. Like the CatSper1, CatSper2, and CatSper3, the inner LWI is more conserved than the outer LWI in CatSper4. Similarly, Seq2Logo analysis was performed for the twelve individual LWI regions of CatSper2-4. The data indicate that the LWI regions are not conserved (Fig. 5e). However, position-specific enrichment of aromatic amino acids, primarily Phenylalanine and Tryptophan residues are notable (Fig. 5e).

### Amino Acids Present in the Lipid-Water-Interface Show no Conservation

We calculated the frequencies of amino acids present in the LWI region (Fig S1–4). The frequencies of different amino acids were plotted for total, inner, and outer LWI regions. The apparent lack of conservation was noticeable in the frequency plot too. Also, the absence of CatSper1 in birds and amphibians allowed us to compare the frequencies from fishes, reptiles, and mammals only (Fig S1). Similarly, in amphibians, all other subunits (i.e. CatSper2-4) remain missing (Fig S2-4).

In the case of CatSper1, the frequencies of Phenylalanine and Leucine are higher at the inner LWI as compared to the outer LWI in all the taxon analyzed here. Also, the frequencies of Phenylalanine and Leucine are higher than the natural abundance of these two amino acids, suggesting a true positive selection of these two amino acids, especially at the inner LWI. Notably, the frequency of Leucine is low in the outer LWI region and lower than the natural abundance, suggesting a true negative selection there. The frequencies of Lysin and Proline are lower than the natural abundance of these amino acids in total, inner and outer LWI regions. Notably, the frequency of Cysteine remains nil in outer as well as almost nil in inner LWI regions, suggesting that Cysteine residue is excluded from the LWI regions of CatSper1 (Fig S1).

A similar analysis was performed for CatSper 2–4 (Fig S2-4). In contrast to CatSper1, in CatSper2, the frequency of Phenylalanine (and not Leucine) is higher than the natural abundance, especially, at the inner LWI (Fig S2). Although the frequencies of Phenylalanine and Leucine are higher at the inner LWI than the outer LWI, a trend that matches with CatSper1. The frequencies of Glutamine and Proline are higher at the outer LWI than the inner LWI and also lower than the natural abundance in the inner LWI region. In CatSper2, the frequency pattern of Cysteine matches well with CatSper1, suggesting that Cysteine residue is excluded from the LWI regions of CatSper2 also.

In the case of CatSper3, the frequency of Phenylalanine is similar to their natural abundance but other aromatic amino acids like Tyrosine, and Tryptophan have higher frequencies in inner and outer LWI respectively. In CatSper3 the frequencies of Alanine and Proline are lower than natural abundance in the total and inner LWI. Cysteine was again excluded from the LWI region from CatSper3, just like CatSper1 and 2 (Fig S3).

In the case of CatSper4 the aromatic amino acid Tryptophan has higher frequency in the inner LWI region in all taxa. Other aromatic amino acids like Tyrosine and Phenylalanine are present in higher frequencies in total LWI in all taxon except in fishes. Frequencies of Aspartic acid, and Proline are lower than their natural abundance in the inner LWI. The frequency of Cysteine shows a similar pattern of exclusion which is observed in other subunits (i.e. CatSper1, 2, and 3) (Fig S4).

### The frequency of hydrophobic and hydrophilic amino acids presents in the lipid-water-interface show specific patterns in certain cases

**Fig. 6:**
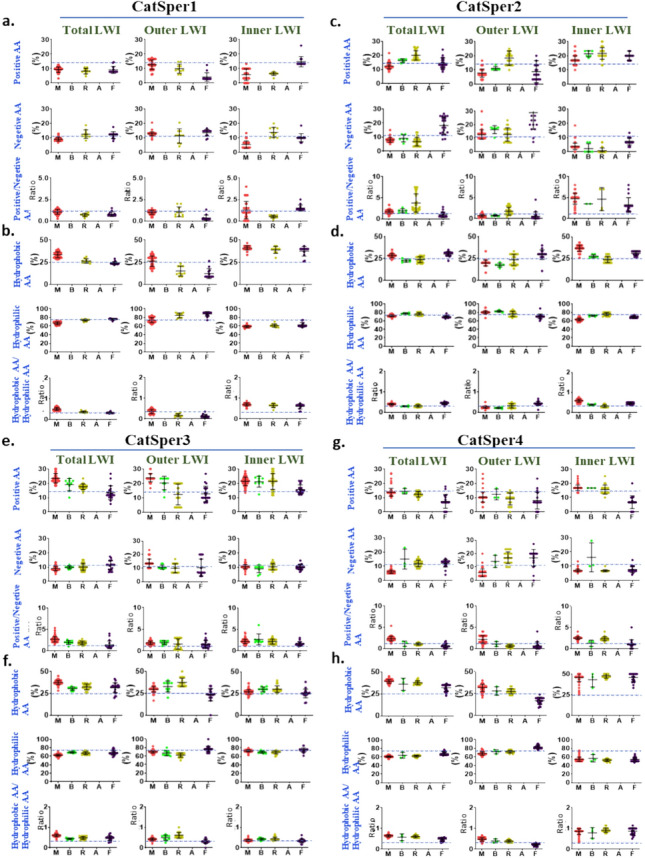
In CatSper1-4, the ratio of positive to negative as well as hydrophobic to hydrophilic residues is not conserved during vertebrate evolution. Values from each species belonging to different vertebrate phyla (fishes: F, amphibians A, reptiles: R, birds: B, and mammals: M) are shown in violet, blue, yellow, green, and red dots, respectively. The total abundance of these amino acids in nature is indicated as a blue dotted line. **a, c, e, and g** The total frequency of positively charged amino acids and negatively charged residues as well as their relative ratio are shown for all CatSper subunits. The frequency for the inner LWI (rightmost) as well as for outer LWI (middle) as well for total (left side) are shown. There is no conservation or pattern observed in the ratio of positive to negative amino acids in the LWI regions. **b, d, f,** and **h** The total frequency of hydrophobic amino acids and hydrophilic residues as well as their relative ratio are shown for all CatSper subunits. The frequencies of the inner LWI (rightmost) as well as for outer LWI (middle) as well for total (left side) are shown. The conserved ratio of hydrophobic to hydrophilic amino acids in the LWI region indicates a selectivity pressure on the overall hydrophobicity in the LWI region

Recently we reported that certain ion channels such as TRPV1 and TRPV4 maintain a specific ratio of total positively charged to total negatively charged residues in their LWI regions (Das et al. 2023; Saha et al. 2017, 2022). Accordingly, we performed a similar analysis for CatSper1-4. We found that in the case of CatSper1, the total positively charged amino acids had a lower frequency in all the taxon than the natural abundance (Fig. 6a). Total negatively charged residues remain variable. Thus, the ratio of positive to negative charged residues remains variable, in inner, outer, and total LWI regions. Similarly, we analyzed the total hydrophobic and total hydrophilic residues of CatSper1. We noted that these frequencies are not conserved. However, at the inner LWI region, the abundance of total hydrophobic residues is more than the natural frequencies and total hydrophilic residues are lower than the natural frequencies (Fig. 6b).

A similar analysis was performed for CatSper2-4 (Fig. 6c–h). Unlike CatSper1, in CatSper2, the frequency of all positively charged amino acids is higher than their natural abundance, and the frequency of all negatively charged amino acids is higher than their natural abundance in the inner LWI. Also, the ratio of all positive to all negative charged residues remains higher in the inner LWI (Fig. 6c). The frequency of all hydrophobic amino acids is higher than the natural abundance in the inner LWI in all taxa (except reptiles) in CatSper2 and this trend is similar to CatSper1 (Fig. 6d). In the case of CatSper3, the frequency of all positively and all negatively charged residues remain variable in total, inner and outer LWI regions. The ratio of positively and negatively charged amino acids also remains variable in all the LWI regions (Fig. 6e). In CatSper3, the frequency of all hydrophobic amino acids is higher than natural abundance in the total and inner LWI regions and this trend matches well with CatSper1 and CatSper2 (Fig. 6f). In the case of CatSper4, the frequency of all positively and all negatively charged amino acids remains variable and their ratio also remains variable in the total, inner, and outer LWI regions (Fig. 6g). In the case of CatSper4, the frequency of all hydrophobic residues remains higher in the inner and total LWI and this trend matches well with other subunits (i.e. CatSper1, 2, 3). In CatSper4, frequency of the all these hydrophilic residues remains low in the inner and total LWI regions. Also, the ratio of hydrophobic to hydrophilic residues is higher at the inner and in total LWI regions and this trend matches well with other sub-units (i.e. CatSper1, 2, 3) (Fig. 6h).

### Full-Length CatSper1-4 Subunits are Less Conserved

As P4 serves as a key molecule/ligand involved in reproduction, we analyzed the conservation of full-length CatSper1, CatSper2, CatSper3, and CatSper4 across the vertebrate species for comparison. We also compared the CatSper proteins with TRPV4, another ion channel that we have reported to be activated by P4 (Dubey et al. 2023). For this purpose, the pairwise distance was calculated using Mega 11.0 and plotted as a boxplot. There is a large diversity in all CatSper across the species. The maximum diversity is observed in the Fish taxon in the case of all the CatSper1-4 units (Fig. 8). The same sub-units remain highly variable in reptilians (but lower than fishes). In mammals, these sub-units are relatively more conserved. In contrast, TRPV4 remains more conserved than any of the CatSper subunits in all the taxon including fishes.

**Fig. 7:**
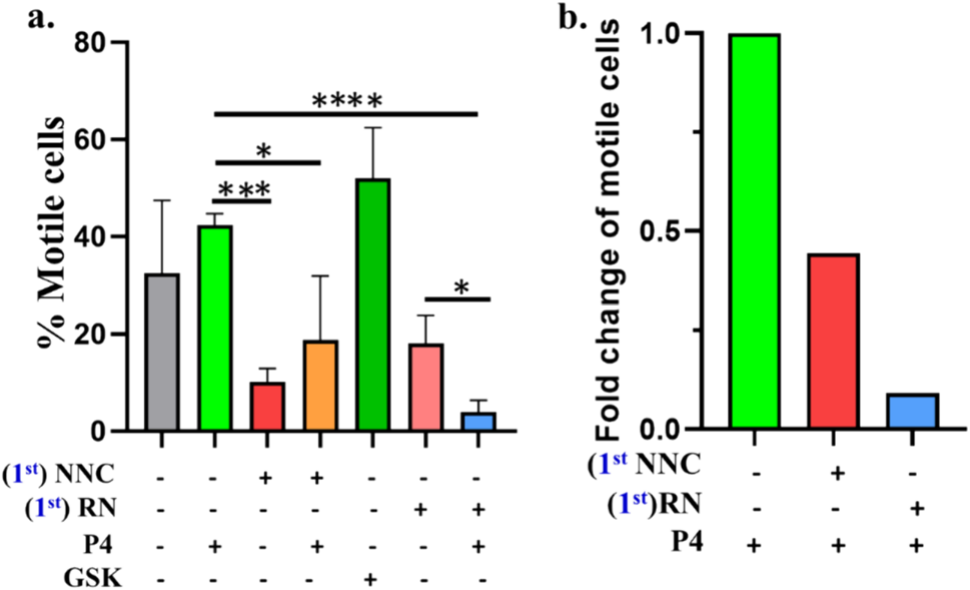
CatSper is not the only ion channel responsible for P4-mediated sperm motility in bull sperm. **a** Bull sperm collected form frozen semen sample were incubated with or without following drugs (NNC, 10 µM; RN1734, 10 µM; P4, 100 nM; GSK, 100 nM) for 1 min and motility was measured in CASA. The percentage of motile sperm were plotted with more than 100 sperm in each condition. In some condition the cells are preincubated for 1 min with drug as mentioned in figure. Test of significance were calculated using Unpaired t-test and P values were calculated by two tailed test with * = p < 0.05, *** = p ≤ 0.0001, **** = p < 0.0001. **b** Fold-change was calculated based on sperm motility in P4-incubated sample (considered as 1). Inhibition of TRPV4 by RN1734 is more effective than inhibition of CatSper by NNC to reduce the P4-mediated motility

**Fig. 8:**
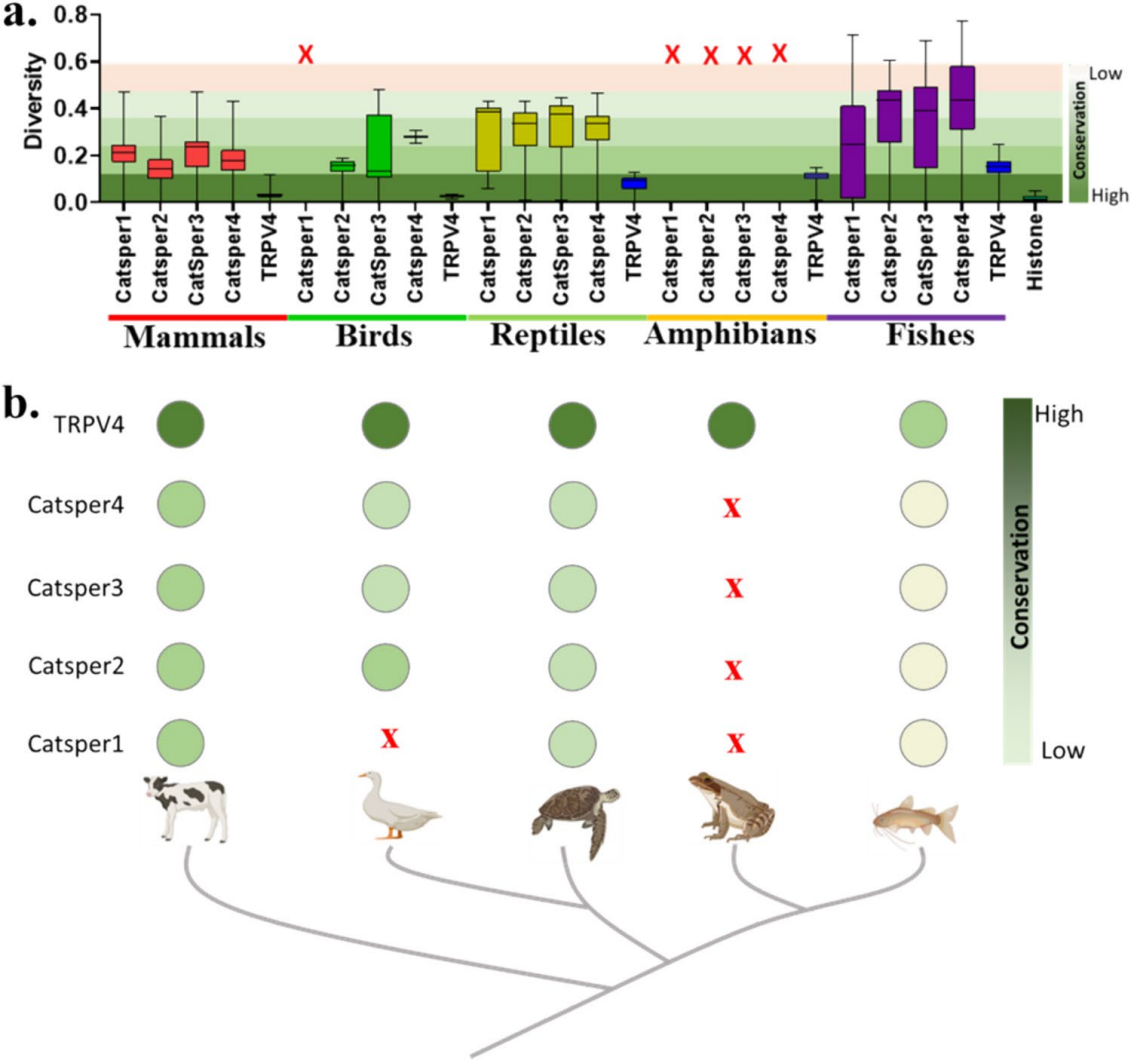
CatSper units are less conserved than TRPV4, another P4-responsive Ca^2+^-ion channel. **a** Shown are the conservation patterns of CatSper1, CatSper2, CatSper3, CatSper4, and TRPV4 for mammals, birds, reptiles, amphibians, and fishes. Higher values indicate less conservation and lesser values indicate higher conservation and are indicated by different background colours. The “X” sign indicates the loss of CatSper unit/s from specific phylogenetic group/s. **b** In birds, only CatSper1 is absent. Similarly, in amphibians, all the CatSper units are absent, suggesting the presence of other proteins that can act as P4-responsive Ca^2+^-ion channels there. TRPV4 is present in all these vertebrate groups and multiple copies of the TRPV4 gene are present in amphibians

Taken together, the data suggest loss-of-gene of certain CatSper subunits in specific taxa, less conservation of full-length CatSper sub-units, and more variability in the LWI regions as compared to the corresponding TM regions. All these suggest that CatSper sub-units are less likely to be the sole P4-responsive molecules present in the sperm cells responsible for reproduction.

### P4 can Increase the Bull Sperm Motility in CatSper-Inhibited Conditions

Sperm motility is an important parameter for the normal physiology of the sperm and it is proposed the CatSper ion channel plays a prominent role in its regulation. It was reported previously that the P4 increases the sperm motility in a Catsper-depended manner (Lishko et al.. 2011). To compare the effect of pharamacological inhibition of CatSper, we performed CASA analysis with bull sample. Here our data indicate that in CatSper-inhibited condition (i.e. NNC-incubated, 10 µM), the motility in bull sperm decreases as compared to P4 (100 nM) incubated conditions (Fig. 7). However, the motility increases further (although non-significantly) when the sperm cells are stimulated by P4 in the CatSper-pre-inhibited condition. This suggests that the inhibition of the CatSper by NNC is insufficient to block the P4-medaited motility regulation, even the inhibitor is used at high concentration (10 µM). This may suggest the involvement of other Ca^2+^ ion channels which could also be activated by P4. Recently we have reported that TRPV4 channel can be activated by P4 and TRPV4 is also present in mature sperm from all the vertebrates (Dubey et al. 2023; Kumar et al. 2016). To check for the role of the TRPV4 ion channel in motility, sperm motility by TRPV4 modulation was also analysed. In the presence of a TRPV4 activator (GSK101, 100 nM), the motility increases significantly and in the presence of a TRPV4 inhibitor (RN, 10 µM), the motility decreases. Notably, in the presence of TRPV4 inhibitor, further application of P4 does not increase the motility further (in fact decreases). Further analysis suggests that pharmacological pre-incubation of CatSper inhibition causes ~ 56% decrease in the motility (Fig. 7b). Comparatively, pharmacological pre-incubation of TRPV4 inhibition causes ~ 90% decrease in the motility. As a proof-of-concept, this data suggest that TRPV4 inhibition is more effective than CatSper inhibition, at least in bull sample.

## Discussion

In this work, we analyzed the presence or absence of core CatSper subunits in the genome of 523 vertebrates, analyzed multiple protein sequences (98 for CatSper1, 137 for CatSper2, 150 for CatSper3 and 137 for CatSper4), conservation of different domains and functional units. The collective data points out specific events during vertebrate evolution, such as loss-of-gene for CatSper subunit/s in certain phylogenetic clades, length-variations, sequence diversification, possible post-translational modifications and regulations, and also the possibilities of involvement of other ion channels for similar functions.

### “Gene-Loss” of CatSper Subunits and Possible Explanations

We noted that amphibians have lost all CatSper1-4 subunits and birds have lost only CatSper1 subunit. Notably, the core “CatSpermasome” depicts hetero-tetramer and is made of one copy of each CatSper1, CatSper2 CatSper3, and CatSper4 subunits. The core “CatSpermasome” structure derived from the mouse indicates that it is a tetramer and it lacks the “symmetry”, which is a common feature of canonical VGIC structure (Clapham and Hulse 2021; de Lera and Kraus 2015; Zhang et al. 2018). Thus, hetero-tetramers forming the functional CatSpermasome are found to be “asymmetric” in nature, both in terms of protein length and also by the sequence. Indeed, our analysis for full-length protein sizes indicates that the core-forming heterotetramer, i.e. CatSperα has maintained the asymmetry in all vertebrates. Such asymmetry is maximum in mammals, but also significant in all other species. In this context, it is worth mentioning that the CatSper1 sequence is approximately double in size that of CatSper2, CatSper3, or CatSsper4 in mammals. Thus, it is not clear how loss-of-gene for one or more subunits will affect the organization of functional/core CatSpermasome. Consequently, it might be possible that in cases of “gene-loss”, other sub-units compensate for the deficit or completely another set of protein/s compensates for the critical functions mediated by CatSpermasome.

We noted that the length of CatSper1 is highly variable among all vertebrates. Our data indicate that the CatSper1 of mammals has a longer N-terminal and a relatively shorter C-terminal. This large N-terminal domain of CatSper1 (as observed in most mammals) is suggested to have a pH-sensing function/s due to the high Histidine content (Ren et al. 2001). We also observed that the pore-forming units of TM5 and TM6 are more conserved than the voltage-sensing domain made of TM1, TM2, TM3, and TM4 in CatSperα1-4. The “pore-region” of CatSper, which contains the “selectivity filter” is fairly and more conserved than the full-length CatSper. The differential conservation might indicate the differential evolutionary selection pressure on the distinct functional/structural units. Notably, the selection pressure seems to be more on the core CatSper pore-forming units than the voltage-sensing units. The loop regions also remain variable by a few amino acids, and such small variations in the loop can also be critical and seems to be specific for certain phylogenetic groups. Though the critical residues contributing to the selectivity filter are not well understood, such residues can be identified from the conservation and molecular selection events and could be analyzed further in the future. Collectively, this analysis indicates that during vertebrate evolution, the gross external macro-environment, membrane micro-environments, and/or the ligands affecting CatSper function/s remain variable, can also be species-specific, and adaptation to this variability seems to be critical for successful fertilization by the sperm cells from different species.

### LWI Regions Remain Variable in all Catasper1-4 Sub-Units

Since hydrophobicity is a very important property in determining the structure of the channel, functional interaction, and orientation in the lipid membrane, we analyzed all hydrophobic and hydrophilic amino acids present in the LWI regions (Simm et al. 2016). We found that for all CatSper1-4 subunits, the inner LWI regions are relatively less diverged than the outer LWI regions. The inner LWI regions have higher contents of hydrophobic residues than the hydrophilic residues. The distribution of specific amino acids allowed us to understand the possible nature of “the ligand/s and the ligand-binding regions”. Notably, the exact P4-binding region present in the CatSper is not known yet, but P4 is known to be enriched in the LWI regions. This may suggest that the inner LWI residues act as a potential binding site for P4. Thus, it is expected that P4 interacts with the sequence stretches/residues present in the LWI regions. In our analysis, we could not find any specific/conserved sequence stretch (neither in the outer nor in the inner LWI region) that suggests positive molecular selection due to single/specific ligand-binding. Thus, the data indicate two key possibilities: first, regulation of CatSper by variable ligands rather than a single ligand. Second, the high hydrophobic nature of the ligand/s, such as lipids and/or lipids-derived products which preferably act on the inner LWI region. This accords well with the reports demonstrating that apart from P4, other lipid derivatives such as prostaglandins also act as modulators of the CatSper. Regulation of CatSper by other lipids can also be relevant for the reproductive success of vertebrates with external fertilization.

### Possible Regulation of CatSper1-4 by Different Post-Translation Modification

The frequency analysis of all these sub-units across different phylogenetic groups indicates important issues of possible regulation of CatSper1-4 subunits by post-translational modifications. First, we analyzed the Arginine and Asparagine residues present on the outer LWI region for possible N-linked glycosylation. Similarly, we also analyzed the presence of Serine, Threonine, and Tyrosine for possible O-linked glycosylation. We noted neither conservation nor any specific pattern for these residues in the outer LWI regions. In addition, these residues are not abundant above their natural frequencies in the outer LWI regions. This may indicate a lack of conserved position and/or residue dedicated to glycosylation. In other words, the glycosylation possibilities of CatSper1-4 remain variable during vertebrate evolution.

The post-translational modification of Cysteine residues is important for cell signaling and protein stabilization. Also, the Cysteine dimerization is very important for protein secondary structure stabilization (Li et al. 2021). Cysteine modification of ion channels, especially at the loop or TM regions restricts the channel’s conformational flexibility leading to irreversible changes in the channel properties. We observed that the CatSperα1-4 has almost excluded the Cysteine residues at the LWI region, both from the inner as well as outer LWI region. In this regard, the near exclusion of Cysteine from all the LWI regions of CatSper1-4 during the vertebrate evolution (i.e. 440 MY) strongly indicates that the possible occurrence of Cysteine residues in the LWI region will probably face Cysteine modification that might provide deleterious functions to the sperm. This accords well with the fact that sperm cells are subject to high ROS-signaling which can potentially cause Cysteine modification (Chianese and Pierantoni. 2021).

We also observed that in the inner LWI region in CatSper1, the frequency of Serine is high and retained well above the natural abundance, especially in mammals. In contrast, the frequencies of Threonine and Tyrosine remain variable and not higher than natural abundance. As Serine acts as an important site for phosphorylation by Serine-Threonine kinases (such as PKCs), the data strongly suggest that Serine residues present in the inner LWI region of vertebrates are possible targets of Serine-Threonine kinases. In this regard, we demonstrate that the serine residue present in the N-TM5 is conserved in all vertebrates and this can be a possible site for protein phosphorylation. In specific, Ser579 (as per human CatSper1) is a strong candidate for phosphorylation. In silico analysis of the 18 amino acid stretch sequence (representing loop sequence between TM4 and TM5) of human CatSper1 also suggests possible phosphorylation by at least PKCs/Cdc2 (possible other kinases also) (data not shown). The importance of this Serine residue in the regulatory function of CatSper1 can be explored in the future.

### Regulation of CatSper1-4 by Snorkeling Amino Acids

The relative positioning of ion channels in the lipid bilayer is an important parameter for their effective function. In this context, not only the occurrence but also the positioning of aromatic amino acids is critical. Aromatic amino acids located at the lipid-water-interface are known to act as an anchor of the ion channels due to the possible snorkeling effect and their ability to form hydrogen bonds with lipids as previously reported (Adamian et al. 2005; De Planque et al. 2002). Also, there are other snorkeling amino acids such as Arginine which has more occurrence than the natural frequency in CatSper2 (only in the inner LWI region) and CatSper3 (both in inner and outer LWI). Also, lysine which is another snorkeling amino acid was analysed but no discernable pattern of conservation was detected. All these snorkeling amino acids help to adjust the ion channel properly along the z-axis in the lipid membrane. In this context, our data suggest that the frequencies of aromatic amino acids especially in the inner LWI region were higher than their natural abundance in all the CatSperα. Higher frequencies were observed for residues with aromatic side chains (like Phenylalanine and Tryptophan) at the inner LWI region. Such a higher occurrence of these residues can provide a better snorkeling effect and help in the protein orientation in the lipid membrane. We also found that the frequencies of Proline residue are low on the inner LWI region in all CatSperα sub-units. Since the Proline provides rigidity to the protein secondary structure, we postulate that the lower frequency of Proline residues at the LWI region is part of a molecular adaptation of the CatSper protein to provide better flexibility in the inner LWI region. Notably, the frequency of total hydrophobic amino acids is higher in the LWI region when compared to the natural abundance of amino acids.

### Possible Role of CatSper Sub-Units in Other Functions

Notably, CatSper1 knockout homozygous female mice (*CatSper1*^*−/−*^) are fully fertile (Ren et al. 2001). CatSper1 heterozygous male (*CatSper1*^±^) is also fully fertile and develops mature sperm cells (Ren et al. 2001). Only CatSper1 knockout homozygous male (*CatSper1*^*−/−*^) is sterile. So far, involvement of CatSper is highlighted for its role mainly in “hyper activation” in mammalian sperm. Hyperactivation is a complex process where multiple factors are involved. Recent findings also suggest that CatSper2-induced Ca^2+^-influx is not essential for sperm rotation (rheotaxix) (Schiffer et al. 2020). So far it is also not known if total knock out of CatSper alters the proteome of mature sperms or affects the expression and localization of other ion channels or some key factors. Notably, the CatSper1 knock out male has a slightly bigger testis and more sperm count, suggesting that CatSper may also be involved in other processes as well (Ren et al. 2001). Indeed, recent findings suggest that CatSper is also involved in certain forms of Cancer (Huang et al. 2022; Meng et al. 2022). Catsper2 is also involved in deafness (Deafness-Infertility-Syndrome, DIS) suggesting that the functions of different subunits are not limited to sperm cells only. It might also be possible that different CatSper subunits are expressed in different tissues and may have different sub-cellular localization and functions (Hildebrand et al. 2010; Zhang et al. 2007).

### Comparatives of CatSper with Other P4-Responsive Ca^2+^-Channel Present in Sperm

P4-mediated upregulation in Ca^2+^-level and subsequent physiological changes in the sperm cell is well established, but mainly from experiments conducted on mammalian systems (Lishko et al. 2011). The molecular target/s of P4, i.e. the Ca^2+^ channel/s present in sperm membrane remains a debatable topic. CatSper gene is also present in invertebrates where biological synthesis of P4 is absent (Cai and Clapham. 2008). Despite the crucial role the CatSper ion channel plays in reproduction, the lack of constant selection pressure during vertebrate evolution (~ 440 MY) points that other ion channels may compensate for its action.

Previously we demonstrated that TRPV4 is present in the mature sperm cells from all the vertebrates (Kumar et al. 2016). Recently we demonstrated that TRPV4 can be activated by P4 and can induce Ca^2+^-influx (Dubey et al. 2023). We also demonstrated that P4 as well as a few other steroids interact with the TM4-Loop-TM5 region of TRPV4 (Dubey et al. 2023; Kumari et al. 2015). All these allowed us to compare the different aspects of CatSpermasome with TRPV4, especially in the context of P4-mediated signaling events in sperm cells and molecular selection observed during vertebrate evolution (Fig. 7). For several reasons, we propose that the CatSpermasome is not the sole molecular target that has been selected for P4 response in sperm cells, even within vertebrates. First, at least 1 or more CatSper subunits are lost from certain species, whereas at least one copy of TRPV4 is retained in all the vertebrates. So far there is no vertebrate known where the TRPV4 gene is lost. Second, as a proof-of-concept, there is at least one case (i.e. in *Xenopus laevis* and *Xenopus tropicalis*) where all the CatSper subunits are missing and multiple copies of TRPV4 are present (gene loss vs gene multiplication) (Kumari et al. 2015). Third, TRPV4 is known to form homo-tetramer and the functional structure is highly symmetric. In contrast, the functional CatSper from different species is expected to be highly asymmetric. Fourth, the “sequence length” is highly conserved for TRPV4 (approximately 871 amino acids) whereas the sequence lengths vary a lot in the case of CatSper sub-units. Fifth, the full-length sequence of TRPV4 is much more conserved than any of the CatSper sub-units and in any vertebrate phyla. Sixth, the lipid-water-interface regions, highly flexible yet functionally important segments of ion channels are more conserved for TRPV4 than any of the CatSper sub-units (Das et al. 2023). Seventh, the LWI region of TRPV4 also maintains a specific pattern as supported by conserved ratio values (Hydrophilic to hydrophobic 2.05, positive to negative 6.96) throughout the vertebrate evolution (Das et al. 2023). In contrast, CatSper sub-units do not show any conserved ratio values. Eighth, the LWI regions of TRPV4 are more conserved than the respective TM regions, suggesting more selection pressure on the LWI residues (mostly for ligand-binding and protein functioning) whereas, for CatSper sub-units, the LWI regions are less conserved than the respective TM regions (suggesting more selection at the TM regions than the LWI regions). Ninth, CatSper is present only in 53% of mature sperm cells (Humans) whereas TRPV4 is present almost in all mature sperm cells (Tamburrino et al. 2014). Tenth, qualitative difference in terms of expression and localization is demonstrated for TRPV4 in motile and non-motile human mature sperm. In contrast, so far, no difference in the expression and/or localization of CatSper is shown in motile vs non-motile cells. Eleventh, CatSper sub-units are physically present in the mice sperm, but all P4-mediated functions cannot be blocked by inhibiting CatSper, suggesting that endogenous CatSper is not involved in P4-signaling, at least in mice (Lishko et al. 2011). Twelfth, activation of CatSper needs a relatively higher amount of P4, though at the physiological level (Smith et al. 2013). Notably the relative concentration of progesterone (and other steroids) is more on the lipid water interface region than the hydrophobic core of the lipid membrane (Atkovska et al. 2018; Crowley, et al. 2022). Altogether the data strongly indicate that in the entire vertebrate system, CatSper is not the sole molecular unit responsible for P4-mediated Ca^2+^-influx and/or hyperactivation events. We propose that the CatSper-mediated P4-signalling event is relevant for certain mammals only and not relevant for all vertebrates (or even invertebrates). We also propose that in vertebrates, CatSper can also recognize diverse hydrophobic ligands other than P4. This concept accords well with the fact that CatSper is reported to be activated by PGE1, PGE2, and PGE1α (Lishko et al. 2011). We also propose that TRPV4 or even other ion channels can also act as P4-responsive Cation channel/s in mature sperm cells from different vertebrate origins.

### Limitations of this Study

As CatSper1-4 are poorly conserved and subject to sequence variations, proper alignment of multiple sequences from multiple species was difficult, mainly due to sequence variations and wide gaps. Also, the prediction results remain variable in terms of the number of transmembrane regions when compared to the Uniprot sequence. To avoid these variabilities, we analyzed all the data based on the alignment file and we selected human sequence from Uniport as our reference for different transmembrane regions. Also, the accurate prediction of TM regions by different web servers often gave variable results where both the “length of each TM” as well as the “starting-ending residues” of each TM remain variable (often by 2–3 amino acids or sometimes more). In some cases, the “loop-region” between two adjacent TM regions remains too small to accurately demark the LWI regions. To avoid large-scale variability, we have removed a few sequences from the entire data set. Thus, the data we represent in this work does not represent accurate positioning or universal demarking of the LWI regions applicable to all species. In that context, considering the 5 amino acid stretch makes a better approximation of the positioning of the LWI regions.

## Conclusion

Sperm physiology is regulated by various molecular mechanisms and perfect sperm functions are responsible for reproductive success and thus also for speciation. As reproduction is a highly specific-specific event, retaining certain variations in terms of molecular identity is critical. Such diversity in the specific sets of important proteins involved in reproduction are observed, such as for Izumo1 and Juno (sperm and oocyte fusion factors) and also for TRPA1 (a poly-modal and non-selective cation channel) (Grayson 2015; Saha et al. 2017). In a similar trend, CatSper seems to play a significant role in reproduction success, yet remains highly variable. Our study strongly suggests that overall CatSper remains variable and different parts of the CatSper have gone through different evolutionary pressures. Previously, CatSper was reported to be the most important ion channel of sperm in mammals and responsible for P4-induced sperm capacitation. However, loss of these genes from some taxa, and diversity in protein sequence indicate that CatSper may not be the only P4-responsive cation channel present in the sperm cells and mature sperms might also have other ion channels for similar functions. In future, it might be possible to target other ion channels successfully even in sperm cells from patients with defective CatSper ion channels for in vitro fertilization.

### Supplementary Information

Below is the link to the electronic supplementary material.

Supplementary file1 (PDF 1875 KB)

## Data Availability

All data generated or analyzed in this study are included in this manuscript [and its supplementary information files]. The additional datasets analyzed in the current study can be available from the corresponding author on reasonable request.

## Abbreviations

C-TM: C-terminal transmembrane domain
LWI: Lipid-water-interface
N-TM: N-terminal transmembrane domain
P4: Progesterone
PGE: Prostaglandin
TRPV4: Transient receptor potential cation channel subfamily vanilloid member 4
VGIC: Voltage-gated ion channel

## References

[CR1] Adamian L, Nanda V, DeGrado WF, Liang J (2005) “Empirical lipid propensities of amino acid residues in multispan alpha helical membrane proteins”. proteins: structure. Funct Genet 59:496–509. 10.1002/prot.20456 10.1002/prot.20456 15789404

[CR2] Atkovska K, Klingler J, Oberwinkler J, Keller S, Hub JS (2018) Rationalizing steroid interactions with lipid membranes: conformations, partitioning, and kinetics. ACS Cent Sci 4(9):1155–1165. 10.1021/acscentsci.8b00332 30276248 10.1021/acscentsci.8b00332 PMC6161064

[CR3] Bernhofer M, Kloppmann E, Reeb J, Rost B (2016) “TMSEG: novel prediction of transmembrane helices”. proteins: structure. Funct Bioinform 84:1706–1716. 10.1002/prot.25155 10.1002/prot.25155 PMC5073023 27566436

[CR4] Brown SG, Publicover SJ, Barratt CLR, Sarah J, da Silva M (2019) Human sperm ion channel (Dys)function: implications for fertilization. Hum Reprod Update 25(6):758–776. 10.1093/humupd/dmz032 31665287 10.1093/humupd/dmz032 PMC6847974

[CR5] Cai X, Clapham DE (2008) Evolutionary Genomics Reveals Lineage-Specific Gene Loss and Rapid Evolution of a Sperm-Specific Ion Channel Complex: CatSpers and CatSperβ. PLoS ONE. 10.1371/JOURNAL.PONE.0003569 18974790 10.1371/JOURNAL.PONE.0003569 PMC2572835

[CR6] Chamberlain AK, Lee Y, Kim S, Bowie JU (2004) Snorkeling preferences foster an amino acid composition bias in transmembrane helices. J Mol Biol 339(2):471–479. 10.1016/j.jmb.2004.03.072 15136048 10.1016/j.jmb.2004.03.072

[CR7] Chianese R, Pierantoni R (2021) Mitochondrial reactive oxygen species (ROS) production alters sperm quality. Antioxidants 10(1):1–19 10.3390/antiox10010092 PMC7827812 33440836

[CR8] Clapham DE, Hulse RE (2021) Sperm CatSper ion channel swims into sharper focus. Nature 595:654–655. 10.1038/d41586-021-01945-5 10.1038/d41586-021-01945-5

[CR9] Crowley J, Withana M, Deplazes E (2022) The interaction of steroids with phospholipid bilayers and membranes. Biophys Rev 14(1):163–179 35340606 10.1007/s12551-021-00918-2 PMC8921366

[CR10] Das R, Mohanta S, Dubey NK, Das NK, GoswamiGoswami. C (2023) Human skeletal dysplasia causing L596P-mutant alters the conserved amino acid pattern at the lipid-water-interface of TRPV4. Biochimica Et Biophysica Acta—Biomembranes 1865(2):184085. 10.1016/j.bbamem.2022.184085 36403799 10.1016/j.bbamem.2022.184085

[CR11] de Lera RM, Kraus RL (2015) Voltage-gated sodium channels: structure, function, pharmacology, and clinical indications. J Med Chem 58(18):7093-7118. 10.1021/jm501981g 25927480 10.1021/jm501981g

[CR12] de Planque MR, Boots JW, Rijkers DT, Liskamp RM, Greathouse DV, Killian JA (2002) The effects of hydrophobic mismatch between phosphatidylcholine bilayers and transmembrane α-helical peptides depend on the nature of interfacially exposed aromatic and charged residues. Biochem 41(26):8396–8404. 10.1021/bi0257686 12081488 10.1021/bi0257686

[CR13] Dubey NK, Mishra S, Goswami C (2023) Progesterone interacts with the mutational hot-spot of TRPV4 and acts as a ligand relevant for fast Ca2+-signalling. Biochimica Et Biophysica Acta—Biomembranes 1865(6):184178. 10.1016/j.bbamem.2023.184178 37225030 10.1016/j.bbamem.2023.184178

[CR14] Gervasi MG, Visconti PE (2016) Chang’s meaning of capacitation: a molecular perspective. Mol Reprod Dev 83(10):860–874 27256723 10.1002/mrd.22663

[CR15] Grayson P (2015) Izumo1 and juno: the evolutionary origins and coevolution of essential sperm-egg binding partners. R Soc Open Sci. 10.1098/RSOS.150296 27019721 10.1098/RSOS.150296 PMC4807442

[CR16] Hallgren J, Tsirigos K, Pedersen M, Almagro J, Nielsen H, Krogh A, Winther O (2022) DTU/DeepTMHMM—BioLib. DeepTMHMM Predicts Alpha Beta Transmembrane Proteins Using Deep Neural Netw 50:9

[CR17] Hildebrand MS, Avenarius MR, Fellous M, Zhang Y, Meyer NC, Auer J, Serres C, Kahrizi K, Najmabadi H, Beckmann JS, Smith RJH (2010) Genetic male infertility and mutation of CATSPER ion channels. Eur J Hum Genet 18(11):1178–1184 20648059 10.1038/ejhg.2010.108 PMC2987470

[CR18] Huang Y, Wang Y, Zhongmei Wu, Li T, Li S, Wang C, Ao J, Yang C, Zhou Yu (2022) SOX11-dependent CATSPER1 expression controls colon cancer cell growth through regulation the PI3K/AKT signaling pathway. Genes Genom 44(11):1415–1424. 10.1007/s13258-022-01240-1 10.1007/s13258-022-01240-1 35305240

[CR19] Kito S, Ohta Y (2005) Medium effects on capacitation and sperm penetration through the zona pellucida in inbred BALB/c spermatozoa. Zygote 13(2):145–153. 10.1017/S0967199405003205 16128410 10.1017/S0967199405003205

[CR20] Krogh A, Larsson B, Von Heijne G, Sonnhammer ELL (2001) Predicting transmembrane protein topology with a hidden markov model: application to complete genomes. J Mol Biol 305(3):567–580. 10.1006/jmbi.2000.4315 11152613 10.1006/jmbi.2000.4315

[CR21] Kumar A, Majhi RK, Nirlipta Swain SC, Giri SK, Samanta L, Goswami C (2016) TRPV4 Is endogenously expressed in vertebrate spermatozoa and regulates intracellular calcium in human sperm. Biochem Biophys Res Commun 473(4):781–788. 10.1016/j.bbrc.2016.03.071 27003252 10.1016/j.bbrc.2016.03.071

[CR22] Kumari S, Kumar AA, Sardar P, Yadav M, Majhi RK, Kumar AA, Goswami C (2015) Influence of membrane cholesterol in the molecular evolution and functional regulation of TRPV4. Biochem Biophys Res Commun 456(1):312–319. 10.1016/j.bbrc.2014.11.077 25434996 10.1016/j.bbrc.2014.11.077

[CR23] Kyte J, Doolittle RF (1982) A simple method for displaying the hydropathic character of a protein. J Mol Biol 157(1):105–132. 10.1016/0022-2836(82)90515-0 7108955 10.1016/0022-2836(82)90515-0

[CR24] Li S, Kai Yu, Guandi Wu, Zhang Q, Wang P, Zheng J, Liu ZX, Wang J, Gao X, Cheng H (2021) PCysMod: prediction of multiple cysteine modifications based on deep learning framework. Front Cell Dev Biol 9:617366 33732693 10.3389/fcell.2021.617366 PMC7959776

[CR25] Lin S, Ke M, Zhang Y, Yan Z, Jianping Wu (2021) Structure of a mammalian sperm cation channel complex. Nature 595(7869):746–750. 10.1038/s41586-021-03742-6 34225353 10.1038/s41586-021-03742-6

[CR26] Lishko PV, Botchkina IL, Kirichok Y (2011) Progesterone activates the principal Ca2+ channel of human sperm. Nature 471(7338):387–392. 10.1038/nature09767 21412339 10.1038/nature09767

[CR27] Meng Y, Mulong Du, Dongying Gu, Li C, Li S, Zhang Q, Ben S, Zhu Q, Xin J, Zhang Z, Zhibin Hu, Shen H, Jiang K, Wang M (2022) Genome-wide association analyses identify CATSPERE as a mediator of colorectal cancer susceptibility and progression. Can Res 82(6):986–997. 10.1158/0008-5472.CAN-21-2948 10.1158/0008-5472.CAN-21-2948 35074755

[CR28] Nagy B, Szekeres-Barthó J, Kovács GL, Sulyok E, Farkas B, Várnagy Á, Vértes V, Kovács K, Bódis J (2021) Key to life: physiological role and clinical implications of progesterone. Int J Mol Sci 22(20):22. 10.3390/IJMS222011039 10.3390/IJMS222011039 PMC8538505 34681696

[CR29] Ren D, Navarro B, Perez G, Jackson AC, Hsu S, Shi Q, Tilly JL, Clapham DE (2001) A sperm ion channel required for sperm motility and male fertility. Nature. 10.1038/35098027 11595941 10.1038/35098027 PMC8462998

[CR30] Saha S, Ghosh A, Tiwari N, Kumar A, Kumar A, Goswami C (2017) Preferential selection of arginine at the lipid-water-interface of TRPV1 during vertebrate evolution correlates with its snorkeling behaviour and cholesterol interaction. Sci Rep. 10.1038/s41598-017-16780-w 29196683 10.1038/s41598-017-16780-w PMC5711878

[CR31] Saha S, Mohanta S, Das R, Dalai R, Divyanshi NT, Tiwari A, Kumar A, Goswami C (2022) Ratio of hydrophobic-hydrophilic and positive-negative residues at lipid–water-interface influences surface expression and channel gating of TRPV1. J Membr Biol 255(2–3):319–339. 10.1007/s00232-022-00243-z 35608627 10.1007/s00232-022-00243-z

[CR32] Schiffer C, Rieger S, Brenker C, Young S, Hamzeh H, Wachten D, Tüttelmann F, Albrecht Röpke U, Kaupp B, Wang T, Wagner A, Krallmann C, Kliesch S, Fallnich C, Strünker T (2020) Rotational motion and rheotaxis of human sperm do not require functional CatSper channels and transmembrane Ca^2+^ signaling. EMBO J. 10.15252/EMBJ.2019102363/SUPPL_FILE/EMBJ2019102363-SUP-0017-MOVIEEV16.ZIP 31957048 10.15252/EMBJ.2019102363/SUPPL_FILE/EMBJ2019102363-SUP-0017-MOVIEEV16.ZIP PMC7024840

[CR33] Simm S, Einloft J, Mirus O, Schleiff E (2016) 50 years of amino acid hydrophobicity scales: revisiting the capacity for peptide classification. Biol Res 49(1):1–19. 10.1186/S40659-016-0092-5/FIGURES/8 27378087 10.1186/S40659-016-0092-5/FIGURES/8 PMC4932767

[CR34] Smith JF, Syritsyna O, Fellousc M, Serres C, Mannowetz N, Kirichok Y, Lishko PV (2013) disruption of the principal, progesterone-activated sperm Ca2+ channel in a CatSper2-deficient infertile patient. Proc Natl Acad Sci USA 110(17):6823–6828. 10.1073/pnas.1216588110 23530196 10.1073/pnas.1216588110 PMC3637729

[CR35] Strünker T, Goodwin N, Brenker C, Kashikar ND, Weyand I, Seifert R, Benjamin Kaupp U (2011) The CatSper Channel Mediates Progesterone-Induced Ca2+ Influx in Human Sperm. Nature 471(7338):382–387. 10.1038/NATURE09769 21412338 10.1038/NATURE09769

[CR36] Sun XH, Zhu YY, Wang L, Liu HL, Ling Y, Li ZL, Sun LB (2017) The catsper channel and its roles in male fertility: a systematic review. Reprod Biol Endocrinol 15(1):1–12. 10.1186/s12958-017-0281-2 28810916 10.1186/s12958-017-0281-2 PMC5558725

[CR37] Suzuki R, Fujinoki M (2023) Progesterone increases the success of in vitro fertilization via enhanced sperm hyperactivation in mice. J Reprod Dev 69(3):147–153. 10.1262/jrd.2022-114 36935121 10.1262/jrd.2022-114 PMC10267584

[CR38] Tamburrino L, Marchiani S, Minetti F, Forti G, Muratori M, Baldi E (2014) The CatSper calcium channel in human sperm: relation with motility and involvement in progesterone-induced acrosome reaction. Hum Reprod 29(3):418–428. 10.1093/humrep/det454 24430778 10.1093/humrep/det454

[CR39] Thomsen MCF, Nielsen M (2012) Seq2Logo: a method for construction and visualization of amino acid binding motifs and sequence profiles including sequence weighting, pseudo counts and two-sided representation of amino acid enrichment and depletion. Nucleic Acids Res. 10.1093/nar/gks469 22638583 10.1093/nar/gks469 PMC3394285

[CR40] Wiltbank MC, Souza AH, Carvalho PD, Cunha AP, Giordano JO, Fricke PM, Baez GM, Diskin MG (2014) Physiological and Practical Effects of Progesterone on Reproduction in Dairy Cattle. Animal 8(SUPPL. 1):70–81. 10.1017/S1751731114000585 24703103 10.1017/S1751731114000585

[CR41] Wimley WC, White SH (1996) Experimentally determined hydrophobicity scale for proteins at membrane interfaces. Nat Struct Biol 3(10):842–848 8836100 10.1038/nsb1096-842

[CR42] Zhang Y, Malekpour M, Al-Madani N, Kahrizi K, Zanganeh M, Mohseni M, Mojahedi F, Daneshi A, Najmabadi H, Smith RJH (2007) Sensorineural deafness and male infertility: a contiguous gene deletion syndrome. J Med Genet 44(4):233–240. 10.1136/jmg.2006.045765 17098888 10.1136/jmg.2006.045765 PMC2598039

[CR43] Zhang XC, Yang H, Liu Z, Sun F (2018) Thermodynamics of voltage-gated ion channels. Biophys Rep 4(6):300–319. 10.1007/s41048-018-0074-y 30596139 10.1007/s41048-018-0074-y PMC6276078

