# Supplementary material for: Sperm-Specific CatSper is Not Conserved in All Vertebrates and May Not be the Only Progesterone-Responsive Ion Channel Present in Sperm
Source: J Membr Biol. 2024 Jul 6;257(3-4):215–30. doi: 10.1007/s00232-024-00316-1 (PMC11289002; doi:10.1007/s00232-024-00316-1)
Supplement: Supplementary file 1 — Supplementary file1 (PDF 1875 KB) [file 232_2024_316_MOESM1_ESM.pdf]

Supplementary figures and tables

Sperm-specific CatSper is not conserved in all vertebrates and may not be the only progesterone-responsive ion channel present in sperm

Nishant Kumar Dubey[1,2,*], Vikash Kumar[1,2], Chandan Goswami[1,2, *]

1. National Institute of Science Education and Research Bhubaneswar, School of Biological Sciences, P.O. Jatni, Khurda 752050, Odisha, India.
2. Homi Bhabha National Institute, Training School Complex, Anushakti Nagar, Mumbai 400094, India.

* Correspondence: nishant.dubey@niser.ac.in or chandan@niser.ac.in

Supplementary figures:

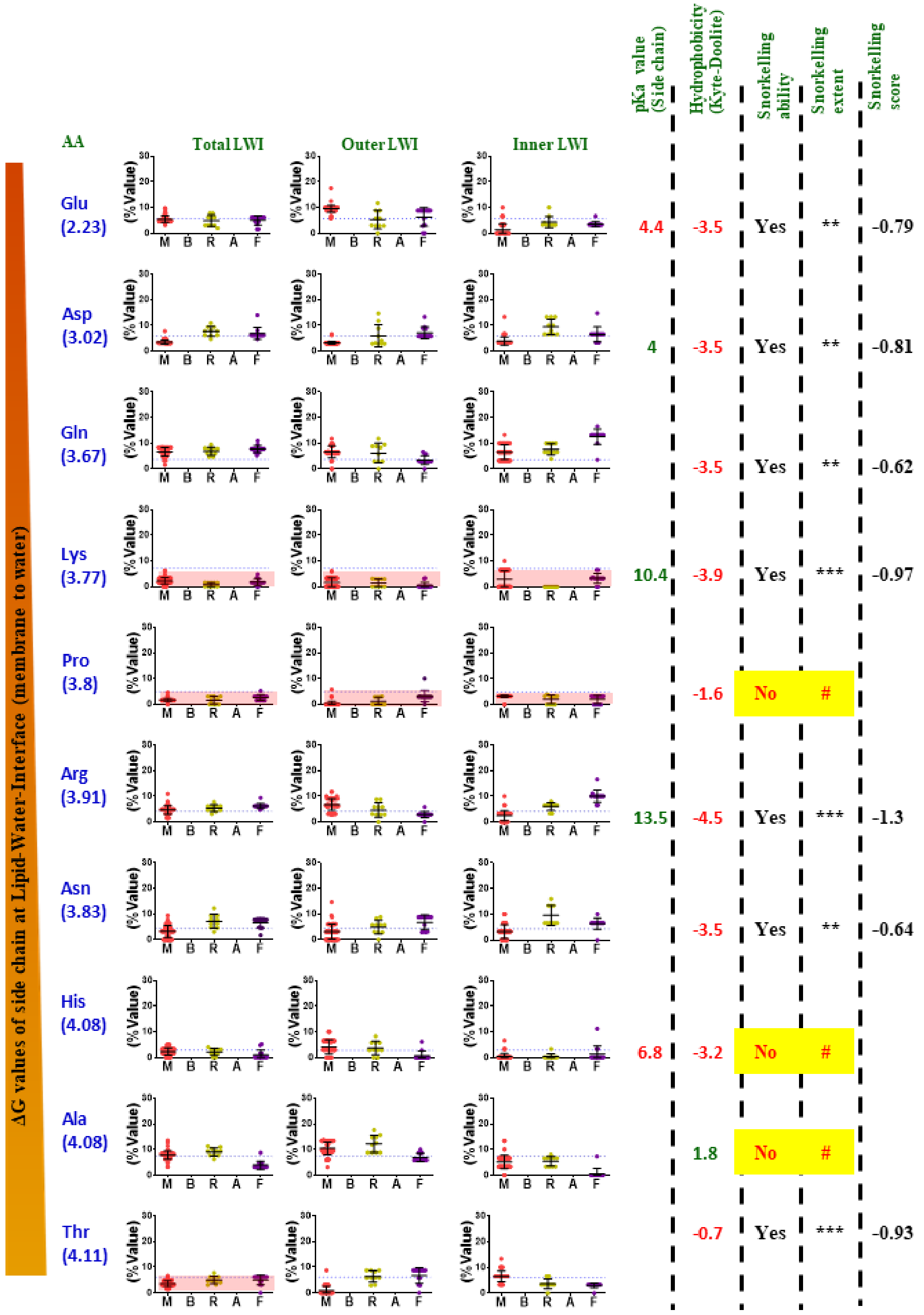

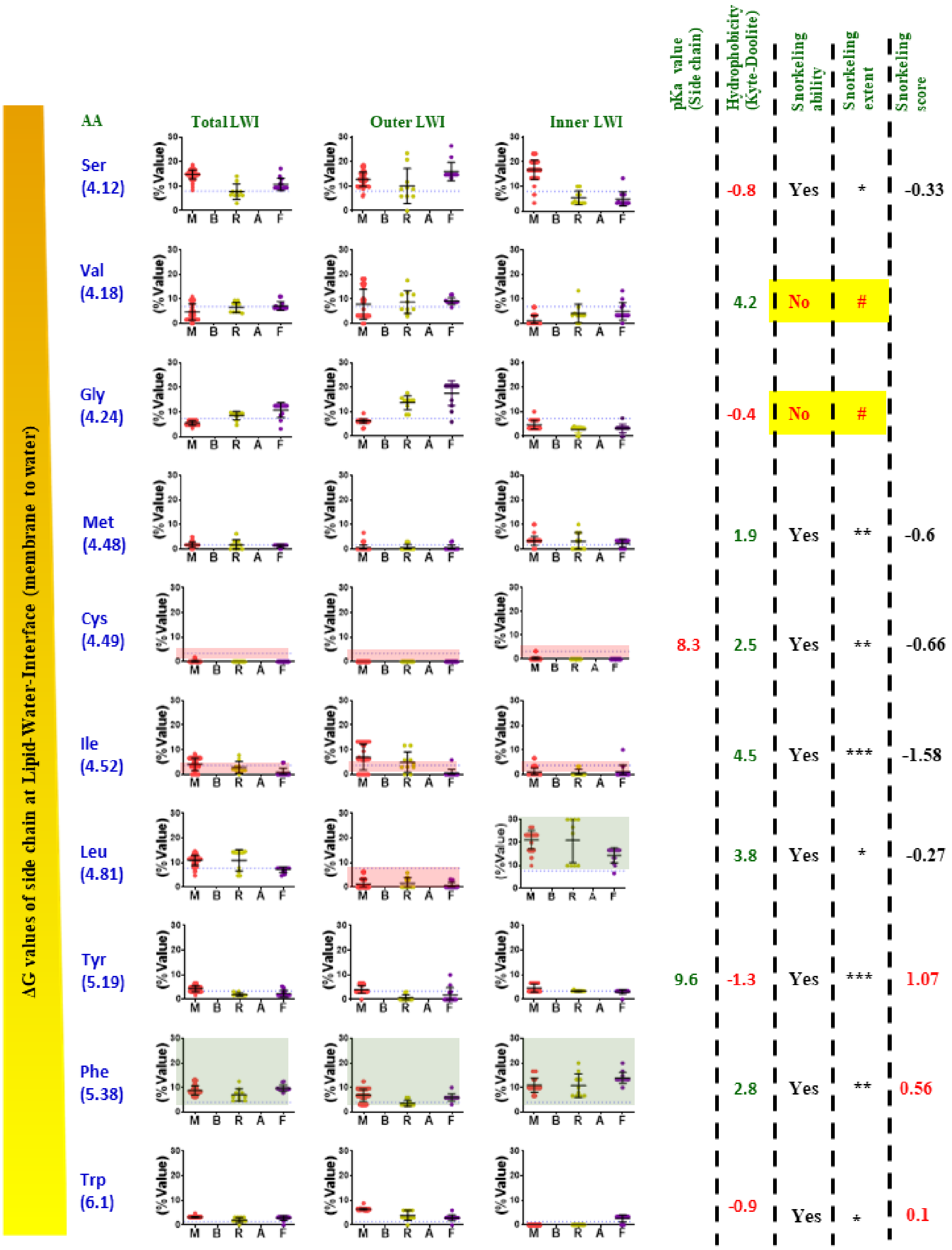

Figure S1: The LWI residues of CatSper1 are not conserved. The Frequencies of individual amino acids present in the LWI region of different phyla are shown. Amino acids at the LWI regions of all 98 species including fishes (F), amphibians (A), reptiles (R), birds (B), and mammals (M) were shown in violet, blue, yellow, green, and red respectively. Their frequency of occurrence was plotted as *Inner LWI* (for the intracellular region, rightmost side), *Outer LWI* (for the extracellular region, middle), and *Total LWI* (left-most side). The amino acids were arranged in increasing order of their ΔG values of side chains. Their pKa values and hydrophobicity were shown on the right side. Amino acids snorkeling propensity was depicted as (***) high, (**) medium, (*) low, and (#) not at all on the right side. The dotted blue line in the individual graph depicts the natural abundance of individual amino acids in nature.

ΔG values of side chain at Lipid-Water-Interface (membrane to water)

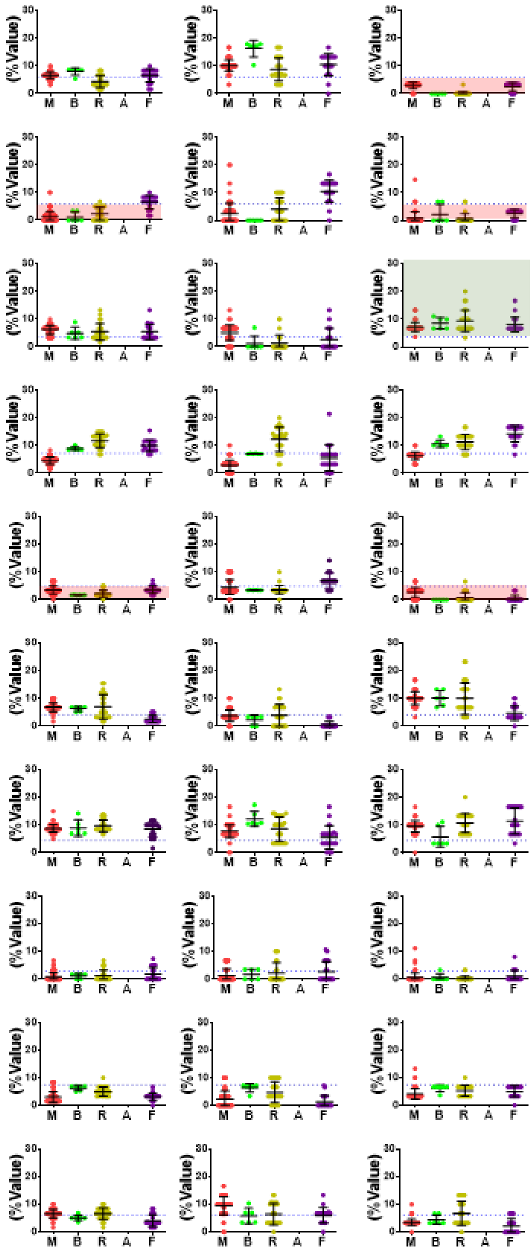

AA	pKa value (Side chain)	Hydrophobicity (Kyte-Doolite)	Snorkelling ability	Snorkelling extent	Snorkelling score
Glu (2.23)	4.4	-3.5	Yes	**	-0.79
Asp (3.02)	4	-3.5	Yes	**	-0.81
Gln (3.67)		-3.5	Yes	**	-0.62
Lys (3.77)	10.4	-3.9	Yes	***	-0.97
Pro (3.8)		-1.6	No	#	
Arg (3.91)	13.5	-4.5	Yes	***	-1.3
Asn (3.83)		-3.5	Yes	**	-0.64
His (4.08)	6.8	-3.2	No	#	
Ala (4.08)		1.8	No	#	
Thr (4.11)		-0.7	Yes	***	-0.93

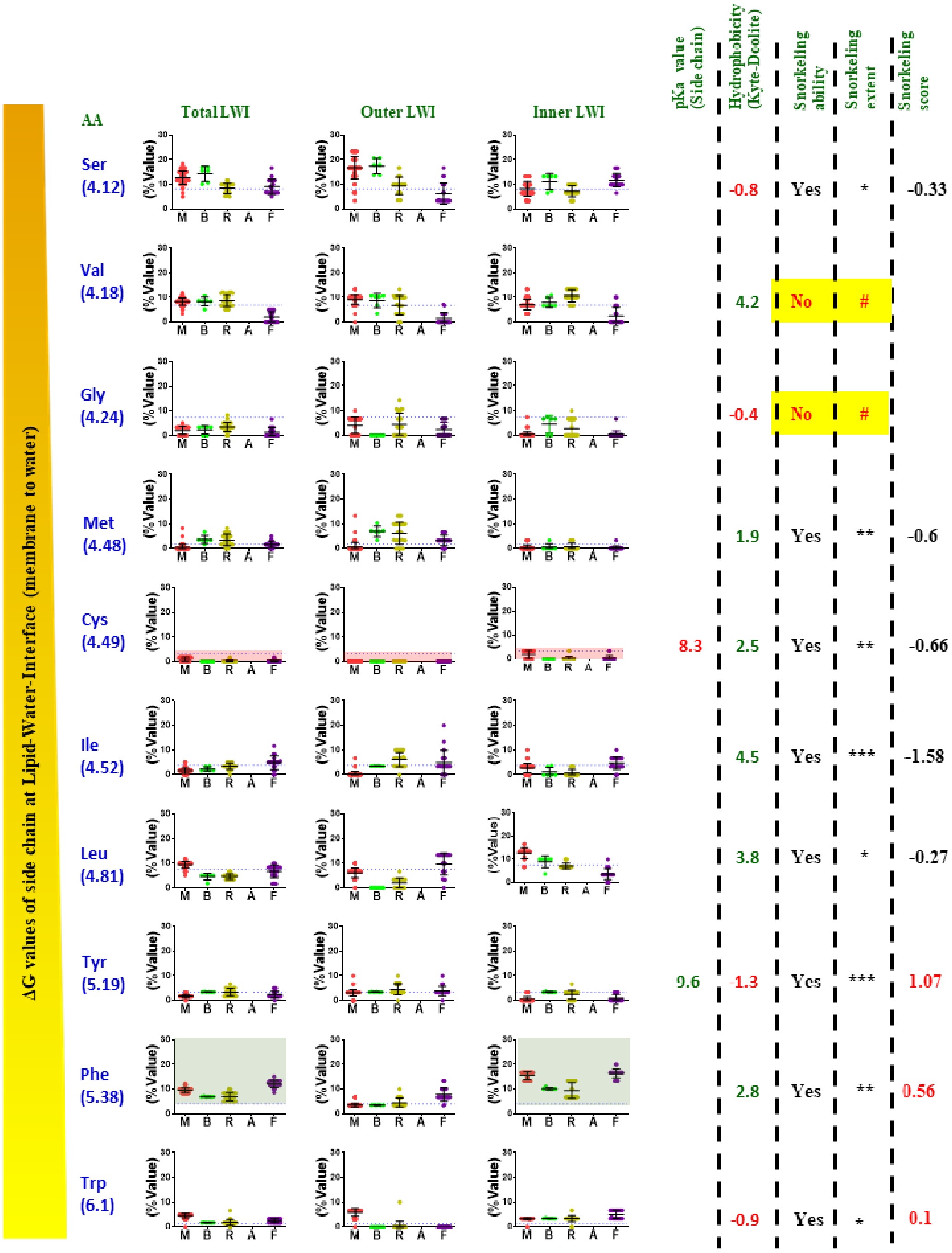

Figure S2: The LWI residues of CatSper2 are not conserved. The Frequencies of individual amino acids present in the LWI region of different phyla are shown. Amino acids at the LWI regions of all 138 species including fishes (F), amphibians (A), reptiles (R), birds (B), and mammals (M) were shown in violet, blue, yellow, green, and red respectively. Their frequency of occurrence was plotted as Inner LWI (for the intracellular region, rightmost side), Outer LWI (for the extracellular region, middle), and Total LWI (left-most side). The amino acids were arranged in increasing order of their ΔG values of side chains. Their pKa values and hydrophobicity were shown on the right side. Amino acids snorkeling propensity was depicted as (***) high, (**) medium, (*) low, and (#) not at all on the right side. The dotted blue line in the individual graph depicts the natural abundance of individual amino acids in nature.

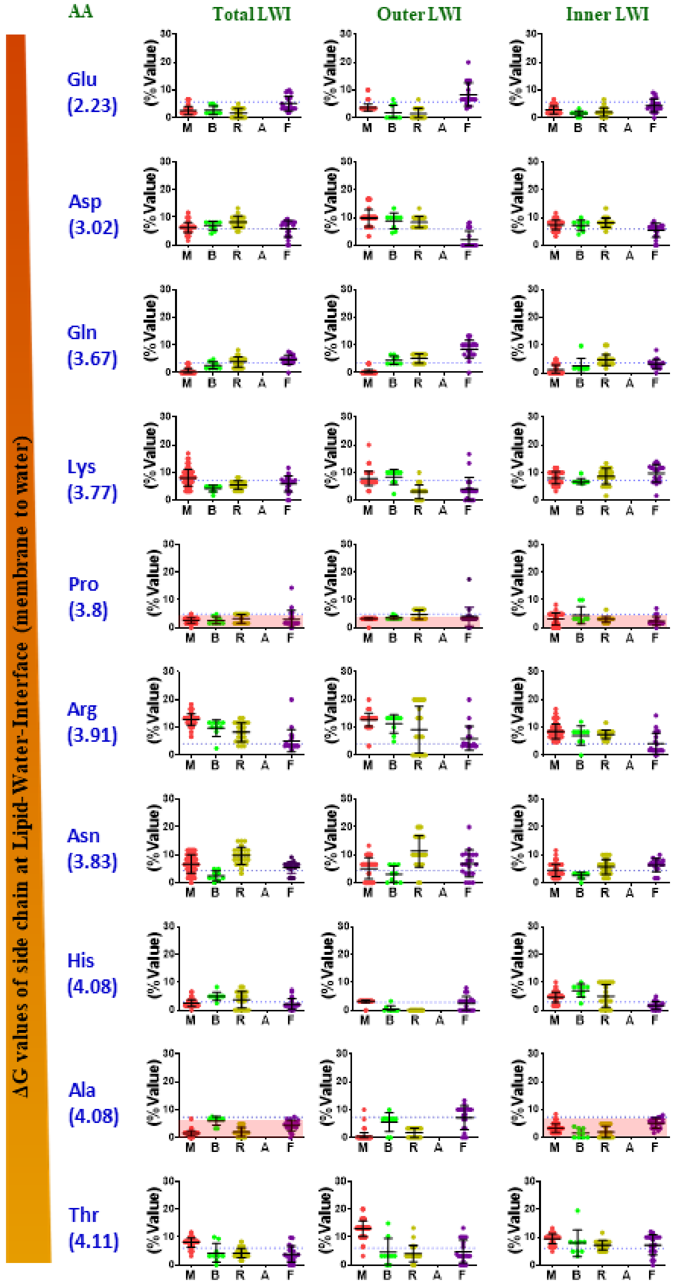

AA	pKa value (Side chain)	Hydrophobicity (Kyte-Doolite)	Snorkelling ability	Snorkelling extent	Snorkelling score
Glu (2.23)	4.4	-3.5	Yes	**	-0.79
Asp (3.02)	4	-3.5	Yes	**	-0.81
Gln (3.67)		-3.5	Yes	**	-0.62
Lys (3.77)	10.4	-3.9	Yes	***	-0.97
Pro (3.8)		-1.6	No	#	
Arg (3.91)	13.5	-4.5	Yes	***	-1.3
Asn (3.83)		-3.5	Yes	**	-0.64
His (4.08)	6.8	-3.2	No	#	
Ala (4.08)		1.8	No	#	
Thr (4.11)		-0.7	Yes	***	-0.93

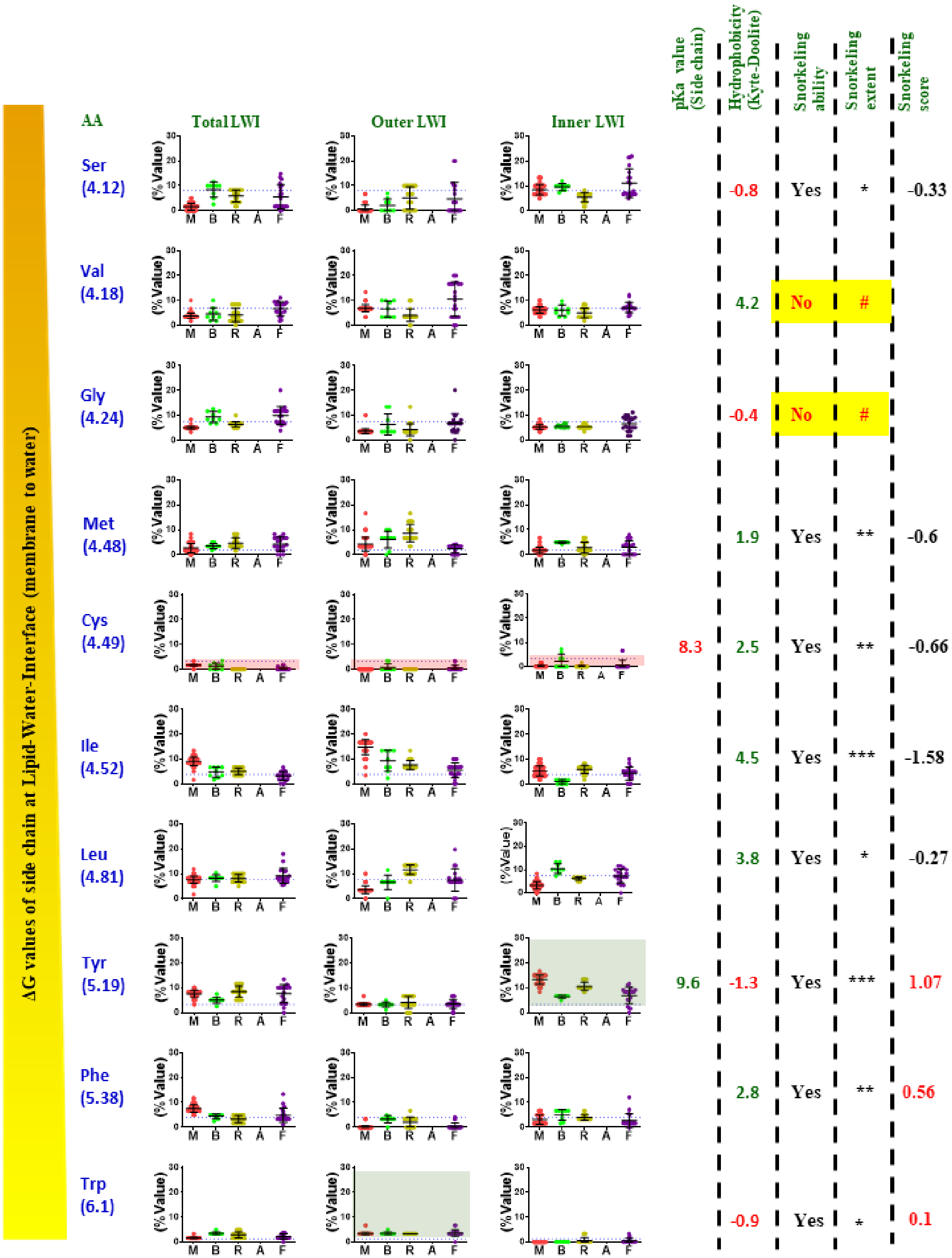

Figure S3: The LWI residues of CatSper3 are not conserved. The Frequencies of individual amino acids present in the LWI region of different phyla are shown. Amino acids at the LWI regions of all 150 species including fishes (F), amphibians (A), reptiles (R), birds (B), and mammals (M) were shown in violet, blue, yellow, green, and red respectively. Their frequency of occurrence was plotted as *Inner LWI* (for the intracellular region, rightmost side), *Outer LWI* (for the extracellular region, middle), and *Total LWI* (left-most side). The amino acids were arranged in increasing order of their ΔG values of side chains. Their pKa values and hydrophobicity were shown on the right side. Amino acids snorkeling propensity was depicted as (***) high, (**) medium, (*) low, and (#) not at all on the right side. The dotted blue line in the individual graph depicts the natural abundance of individual amino acids in nature.

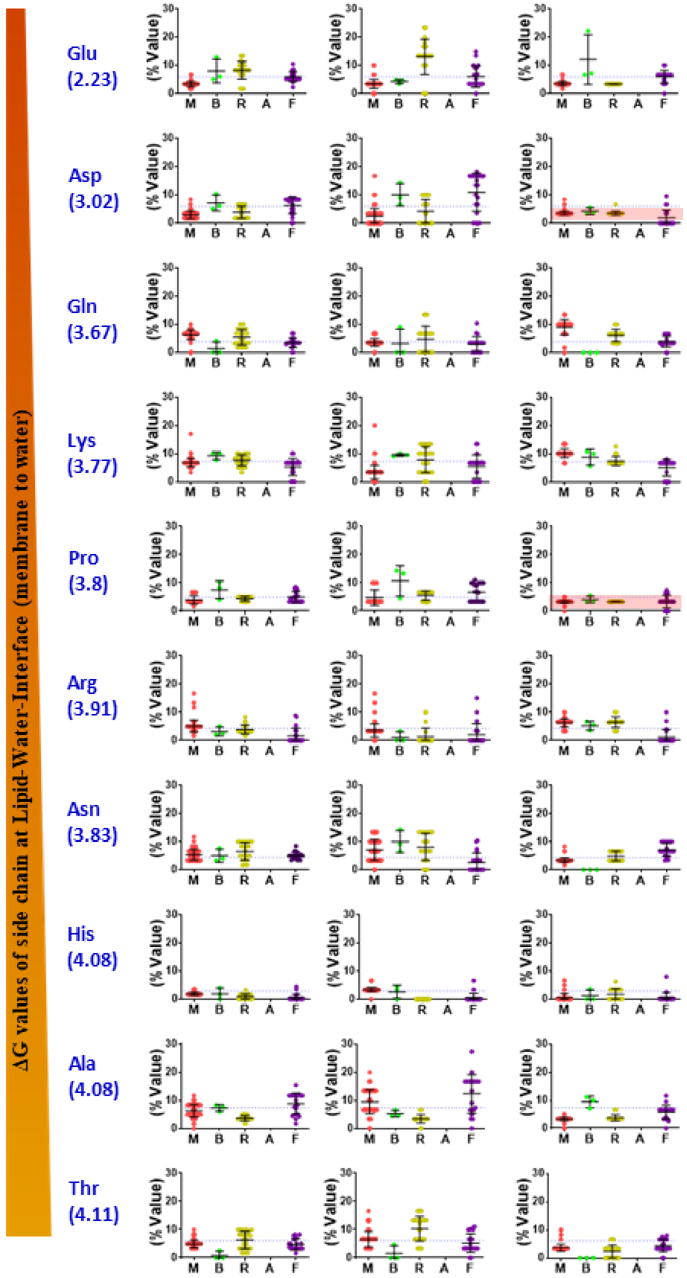

AA	pKa value (Side chain)	Hydrophobicity (Kyte-Doolite)	Snorkelling ability	Snorkelling extent	Snorkelling score
Glu (2.23)	4.4	-3.5	Yes	**	-0.79
Asp (3.02)	4	-3.5	Yes	**	-0.81
Gln (3.67)		-3.5	Yes	**	-0.62
Lys (3.77)	10.4	-3.9	Yes	***	-0.97
Pro (3.8)		-1.6	No	#	
Arg (3.91)	13.5	-4.5	Yes	***	-1.3
Asn (3.83)		-3.5	Yes	**	-0.64
His (4.08)	6.8	-3.2	No	#	
Ala (4.08)		1.8	No	#	
Thr (4.11)		-0.7	Yes	***	-0.93

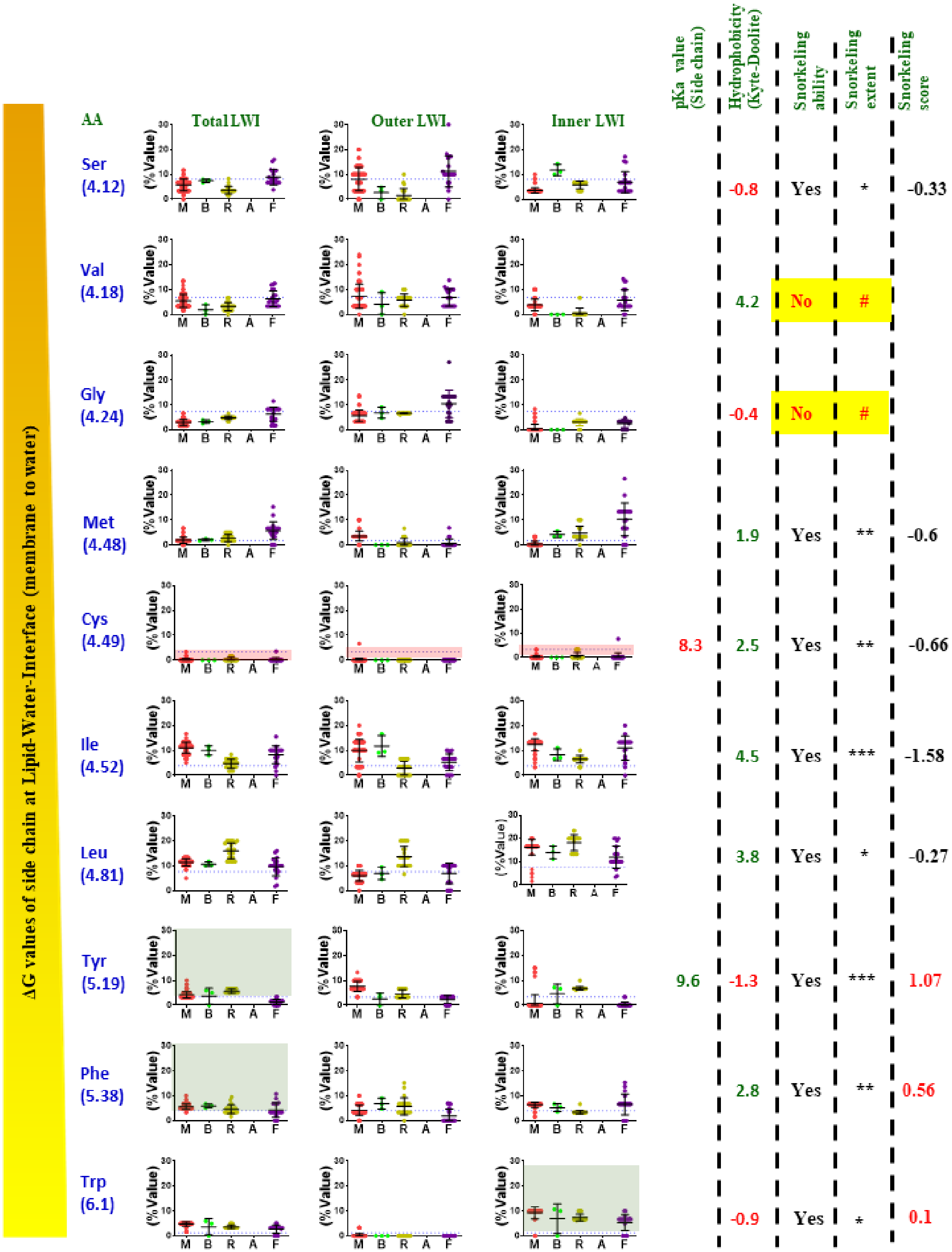

Figure S4: The LWI residues of CatSper4 are not conserved. The Frequencies of individual amino acids present in the LWI region of different phyla are shown. Amino acids at the LWI regions of all 137 species including fishes (F), amphibians (A), reptiles (R), birds (B), and mammals (M) were shown in violet, blue, yellow, green, and red respectively. Their frequency of occurrence was plotted as *Inner LWI* (for the intracellular region, rightmost side), *Outer LWI* (for the extracellular region, middle), and *Total LWI* (left-most side). The amino acids were arranged in increasing order of their ΔG values of side chains. Their pKa values and hydrophobicity were shown on the right side. Amino acids snorkeling propensity was depicted as (***) high, (**) medium, (*) low, and (#) not at all on the right side. The dotted blue line in the individual graph depicts the natural abundance of individual amino acids in nature.

Supplementary tables.

CatSper1			CatSper2		
**Gene ID**	**Species name**	**Class**	**Gene ID**	**Species name**	**Class**
NP_444282.3	*Homo sapiens*	Mammal	NP_001269238.1	*Homo sapiens*	Mammal
KAF6102491.1	*Phyllostomus discolor*	Mammal	XP_024209368.1	*Pan troglodytes*	Mammal
XP_023510249.1	*Equus caballus*	Mammal	XP_008966078.2	*Pan paniscus*	Mammal
XP_039100781.1	*Hyaena hyaena*	Mammal	XP_011890411.1	*Cercocebus atys*	Mammal
XP_040140015.1	*Ictidomys tridecemlineatus*	Mammal	XP_014997573.1	*Macaca mulatta*	Mammal
XP_012633625.1	*Microcebus murinus*	Mammal	XP_009248058.2	*Pongo abelii*	Mammal
XP_038280851.1	*Canis lupus familiaris*	Mammal	XP_033072180.1	*Trachypithecus francoisi*	Mammal
XP_021528699.1	*Aotus nancymaae*	Mammal	XP_010337842.1	*Saimiri boliviensis boliviensis*	Mammal
XP_036991107.1	*Artibeus jamaicensis*	Mammal	XP_032153539.1	*Sapajus apella*	Mammal
XP_025142478.1	*Bubalus bubalis*	Mammal	XP_017401505.1	*Cebus imitator*	Mammal
XP_035119257.1	*Callithrix jacchus*	Mammal	XP_012297576.1	*Aotus nancymaae*	Mammal
XP_025734683.1	*Callorhinus ursinus*	Mammal	XP_002753430.1	*Callithrix jacchus*	Mammal
XP_008059216.1	*Carlito syrichta*	Mammal	XP_008580757.1	*Galeopterus variegatus*	Mammal
XP_037597596.1	*Cebus imitator*	Mammal	XP_008057805.1	*Carlito syrichta*	Mammal
XP_007977056.2	*Chlorocebus sabaeus*	Mammal	NP_001231199.1	*Sus scrofa*	Mammal
XP_004051617.1	*Gorilla gorilla gorilla*	Mammal	XP_001918252.1	*Equus caballus*	Mammal
XP_032009389.1	*Hylobates moloch*	Mammal	XP_014636145.1	*Ceratotherium simum simum*	Mammal
XP_032735402.1	*Lontra canadensis*	Mammal	XP_014702589.1	*Equus asinus*	Mammal
XP_003419462.1	*Loxodonta africana*	Mammal	XP_020011809.1	*Castor canadensis*	Mammal
EHH56064.1	*Macaca fascicularis*	Mammal	XP_039088640.1	*Hyaena hyaena*	Mammal
EHH22670.1	*Macaca mulatta*	Mammal	XP_032273447.1	*Phoca vitulina*	Mammal
XP_011719214.1	*Macaca nemestrina*	Mammal	XP_037010818.1	*Artibeus jamaicensis*	Mammal
XP_036780105.1	*Manis pentadactyla*	Mammal	XP_024588353.1	*Neophocaena asiaeorientalis asiaeorientalis*	Mammal
XP_003274269.2	*Nomascus leucogenys*	Mammal	XP_036910382.1	*Sturnira hondurensis*	Mammal
XP_012392620.1	*Orcinus orca*	Mammal	XP_029093456.1	*Monodon monoceros*	Mammal
XP_003828705.1	*Pan paniscus*	Mammal	XP_004380748.1	*Trichechus manatus latirostris*	Mammal
XP_016776772.1	*Pan troglodytes*	Mammal	XP_004411478.1	*Odobenus rosmarus divergens*	Mammal
XP_003909583.2	*Papio anubis*	Mammal	XP_007467599.1	*Lipotes vexillifer*	Mammal

XP_032497115.1	*Phocoena sinus*	Mammal	XP_034516938.1	*Ailuropoda melanoleuca*	Mammal
XP_007128306.1	*Physeter catodon*	Mammal	XP_034850054.1	*Mirounga leonina*	Mammal
XP_023042194.1	*Piliocolobus tephrosceles*	Mammal	XP_036697628.1	*Balaenoptera musculus*	Mammal
XP_024111251.1	*Pongo abelii*	Mammal	XP_002717986.1	*Oryctolagus cuniculus*	Mammal
XP_010364738.1	*Rhinopithecus roxellana*	Mammal	XP_043411490.1	*Prionailurus bengalensis*	Mammal
XP_003937778.1	*Saimiri boliviensis boliviensis*	Mammal	XP_032337229.1	*Camelus ferus*	Mammal
XP_032138253.1	*Sapajus apella*	Mammal	XP_019790984.2	*Tursiops truncatus*	Mammal
NP_001231186.1	*Sus scrofa*	Mammal	XP_040348453.1	*Puma yagouaroundi*	Mammal
XP_033061807.1	*Trachypithecus francoisi*	Mammal	XP_006932645.1	*Felis catus*	Mammal
XP_041578378.1	*Vulpes lagopus*	Mammal	XP_027813121.1	*Ovis aries*	Mammal
XP_025859734.1	*Vulpes vulpes*	Mammal	XP_017921464.1	*Capra hircus*	Mammal
XP_008531329.1	*Equus przewalskii*	Mammal	XP_006201781.1	*Vicugna pacos*	Mammal
XP_011897003.1	*Cercocebus atys*	Mammal	XP_030698658.1	*Globicephala melas*	Mammal
XP_011797843.1	*Colobus angolensis palliatus*	Mammal	XP_025775561.1	*Puma concolor*	Mammal
XP_014700476.1	*Equus asinus*	Mammal	XP_026922925.1	*Acinonyx jubatus*	Mammal
XP_007462264.1	*Lipotes vexillifer*	Mammal	XP_042798116.1	*Panthera leo*	Mammal
XP_004770036.1	*Mustela putorius furo*	Mammal	XP_027982416.1	*Eumetopias jubatus*	Mammal
XP_012423891.1	*Odobenus rosmarus divergens*	Mammal	XP_041600272.1	*Vulpes lagopus*	Mammal
XP_017712812.1	*Rhinopithecus bieti*	Mammal	NP_694715.2	*Mus musculus*	Mammal
NP_647462.1	*Mus musculus*	Mammal	XP_031227522.1	*Mastomys couch*	Mammal
XP_028626843.1	*Grammomys surdaster*	Mammal	XP_021048681.1	*Mus pahari*	Mammal
XP_032747026.1	*Rattus rattus*	Mammal	XP_034351659.1	*Arvicanthis niloticus*	Mammal
XP_021079774.2	*Mesocricetus auratus*	Mammal	NP_001012220.1	*Rattus norvegicus*	Mammal
ERE77857.1	*Cricetulus griseus*	Mammal	XP_032759951.1	*Rattus rattus*	Mammal
XP_020020573.1	*Castor canadensis*	Mammal	XP_037060343.1	*Peromyscus leucopus*	Mammal
XP_008271712.1	*Oryctolagus cuniculus*	Mammal	XP_040602085.1	*Mesocricetus auratus*	Mammal
XP_014648643.1	*Ceratotherium simum simum*	Mammal	XP_041534676.1	*Microtus oregoni*	Mammal
XP_015358529.1	*Marmota marmota marmota*	Mammal	XP_027781565.1	*Marmota flaviventris*	Mammal
XP_036851374.1	*Manis javanica*	Mammal	XP_015335591.1	*Marmota marmota marmota*	Mammal
XP_004596899.1	*Ochotona princeps*	Mammal	XP_012865749.1	*Dipodomys ordii*	Mammal
XP_027973773.1	*Eumetopias jubatus*	Mammal	XP_026247569.1	*Urocitellus parryii*	Mammal
XP_030689885.1	*Globicephala melas*	Mammal	XP_040130163.1	*Ictidomys tridecemlineatus*	Mammal
XP_030891359.1	*Leptonychotes weddellii*	Mammal	XP_012622105.1	*Microcebus murinus*	Mammal

XP_033717631.1	*Tursiops truncatus*	Mammal	ELR58640.1	*Bos mutus*	Mammal
XP_026945682.1	*Lagenorhynchus obliquidens*	Mammal	NP_001179406.1	*Bos taurus*	Mammal
XP_021540939.1	*Neomonachus schauinslandi*	Mammal	XP_019500395.1	*Hipposideros armiger*	Mammal
XP_023593887.1	*Trichechus manatus latirostris*	Mammal	XP_010834460.1	*Bison bison bison*	Mammal
XP_043438516.1	*Prionailurus bengalensis*	Mammal	XP_028351175.1	*Physeter catodon*	Mammal
XP_026901306.1	*Acinonyx jubatus*	Mammal	XP_025127616.1	*Bubalus bubalis*	Mammal
XP_029065249.1	*Monodon monoceros*	Mammal	XP_040476457.1	*Ursus maritimus*	Mammal
VFV39208.1	*Lynx pardinus*	Mammal	XP_026335637.1	*Ursus arctos horribilis*	Mammal
XP_042814142.1	*Panthera tigris*	Mammal	XP_024423672.1	*Desmodus rotundus*	Mammal
XP_042761053.1	*Panthera leo*	Mammal	XP_040825145.1	*Ochotona curzoniae*	Mammal
XP_037695357.1	*Choloepus didactylus*	Mammal	XP_012504593.1	*Propithecus coquereli*	Mammal
EQB77702.1	*Camelus ferus*	Mammal	XP_012583994.1	*Condylura cristata*	Mammal
XP_007502734.1	*Monodelphis domestica*	Mammal	XP_012783433.1	*Ochotona princeps*	Mammal
KAF6464301.1	*Rousettus aegyptiacus*	Mammal	XP_021551421.1	*Neomonachus schauinslandi*	Mammal
EMP25853.1	*Chelonia mydas*	Reptile	XP_038606511.1	*Tachyglossus aculeatus*	Mammal
XP_043374601.1	*Dermochelys coriacea*	Reptile	XP_028927144.1	*Ornithorhynchus anatinus*	Mammal
XP_030424853.1	*Gopherus evgoodei*	Reptile	XP_027725730.1	*Vombatus ursinus*	Mammal
XP_032654780.1	*Chelonoidis abingdonii*	Reptile	XP_004466800.2	*Dasypus novemcinctus*	Mammal
XP_025037975.1	*Pelodiscus sinensis*	Reptile	XP_030740367.1	*Echinops telfairi*	Mammal
KAG6927554.1	*Chelydra serpentina*	Reptile	XP_032712526.1	*Lontra canadensis*	Mammal
XP_025029460.1	*Python bivittatus*	Reptile	XP_032199421.1	*Mustela erminea*	Mammal
XP_042294852.1	*Sceloporus undulatus*	Reptile	XP_003756106.1	*Sarcophilus harrisii*	Mammal
XP_039222189.1	*Crotalus tigris*	Reptile	XP_036299774.1	*Pipistrellus kuhlii*	Mammal
XP_034954746.1	*Zootoca vivipara*	Reptile	XP_025896438.1	*Nothoprocta perdicaria*	Bird
XP_029929781.1	*Myripristis murdjan*	Fish	XP_010224371.1	*Tinamus guttatus*	Bird
XP_035627831.1	*Oncorhynchus keta*	Fish	XP_025977010.1	*Dromaius novaehollandiae*	Bird
XP_021446940.1	*Oncorhynchus mykiss*	Fish	XP_013809727.1	*Apteryx mantelli mantelli*	Bird
XP_038821039.1	*Salvelinus namaycush*	Fish	XP_009668422.1	*Struthio camelus australis*	Bird
XP_024253751.2	*Oncorhynchus tshawytscha*	Fish	XP_025943173.1	*Apteryx rowi*	Bird
XP_019904203.1	*Esox lucius*	Fish	XP_026548014.1	*Notechis scutatus*	Reptile
XP_013984536.1	*Salmo salar*	Fish	KAG8133995.1	*E2320_011728 Naja naja*	Reptile

XP_029625636.1	*Salmo trutta*	Fish	XP_013912711.1	*Thamnophis sirtalis*	Reptile
XP_041707145.1	*Coregonus clupeaformis*	Fish	XP_032078221.1	*Thamnophis elegans*	Reptile
XP_041948954.1	*Alosa sapidissima*	Fish	XP_015675657.1	*Protobothrops mucrosquamatus*	Reptile
XP_014339358.1	*Latimeria chalumnae*	Fish	XP_034291914.1	*Pantherophis guttatus*	Reptile
XP_023843765.1	*Salvelinus alpinus*	Fish	XP_039225822.1	*Crotalus tigris*	Reptile
XP_015194281.1	*Lepisosteus oculatus*	Fish	ETE56850.1	*Ophiophagus hannah*	Reptile
			XP_007436523.1	*Python bivittatus*	Reptile
			KAF7237383.1	*Varanus komodoensis*	Reptile
			XP_020660214.1	*Pogona vitticeps*	Reptile
			XP_042300280.1	*Sceloporus undulatus*	Reptile
			XP_028594394.1	*Podarcis muralis*	Reptile
			XP_026582250.1	*Pseudonaja textilis*	Reptile
			XP_034980344.1	*Zootoca vivipara*	Reptile
			XP_003219521.1	*Anolis carolinensis*	Reptile
			XP_015266763.1	*Gekko japonicus*	Reptile
			XP_032650067.1	*Chelonoidis abingdonii*	Reptile
			XP_030434481.1	*Gopherus evgoodei*	Reptile
			XP_042712632.1	*Chrysemys picta bellii*	Reptile
			XP_026504352.1	*Terrapene carolina triunguis*	Reptile
			XP_034640803.1	*Trachemys scripta elegans*	Reptile
			XP_037767429.1	*Chelonia mydas*	Reptile
			KAG6923270.1	*Chelydra serpentina*	Reptile
			XP_019349829.1	*Alligator mississippiensis*	Reptile
			XP_039349139.1	*Mauremys reevesii*	Reptile
			XP_043350092.1	*Dermochelys coriacea*	Reptile
			XP_031658040.1	*Oncorhynchus kisutch*	Fish
			XP_023838872.1	*Salvelinus alpinus*	Fish
			XP_036806913.1	*Oncorhynchus mykiss*	Fish
			XP_038873121.1	*Salvelinus namaycush*	Fish
			XP_029614152.1	*Salmo trutta*	Fish
			XP_013980194.1	*Salmo salar*	Fish
			XP_041692569.1	*Coregonus clupeaformis*	Fish
			XP_019896018.1	*Esox lucius*	Fish
			XP_029524490.1	*Oncorhynchus nerka*	Fish
			XP_015198998.1	*Lepisosteus oculatus*	Fish

			XP_031420931.1	*Clupea harengus*	Fish
			XP_041091045.1	*Polyodon spathula*	Fish
			XP_041966142.1	*Alosa sapidissima*	Fish
			XP_035382788.1	*Electrophorus electricus*	Fish
			XP_038668803.1	*Scyliorhinus canicula*	Fish
			XP_020382095.1	*Rhincodon typus*	Fish
			XP_030621085.1	*Chanos chanos*	Fish
			XP_042199959.1	*Callorhinchus milii*	Fish
			XP_032906080.1	*Amblyraja radiata*	Fish
			XP_036413771.1	*Colossoma macropomum*	Fish
**CatSper3**			**CatSper4**		
**Gene ID**	**Species name**	**Class**	**Gene ID**	**Species name**	**Class**
NP_821138.1	*Homo sapiens*	Mammal	XP_011539734.1	*Homo sapiens*	Mammal
XP_003829312.1	*Pan paniscus*	Mammal	XP_016812240.1	*Pan troglodytes*	Mammal
XP_030867372.1	*Gorilla gorilla gorilla*	Mammal	XP_017711679.1	*Rhinopithecus bieti*	Mammal
XP_003266450.2	*Nomascus leucogenys*	Mammal	XP_003271694.2	*Nomascus leucogenys*	Mammal
XP_032013842.1	*Hylobates moloch*	Mammal	XP_002811283.1	*Pongo abelii*	Mammal
XP_002815944.1	*Pongo abelii*	Mammal	XP_032615286.1	*Hylobates moloch*	Mammal
XP_017727428.1	*Rhinopithecus bieti*	Mammal	XP_003809499.1	*Pan paniscus*	Mammal
XP_008012672.1	*Chlorocebus sabaeus*	Mammal	XP_004025275.2	*Gorilla gorilla gorilla*	Mammal
XP_033037058.1	*Trachypithecus francoisi*	Mammal	XP_035164113.1	*Callithrix jacchus*	Mammal
XP_009447959.1	*Pan troglodytes*	Mammal	XP_010353978.1	*Rhinopithecus roxellana*	Mammal
XP_011824589.1	*Mandrillus leucophaeus*	Mammal	XP_025215380.1	*Theropithecus gelada*	Mammal
XP_003900163.1	*Papio anubis*	Mammal	XP_033083570.1	*Trachypithecus francoisi*	Mammal
XP_025243931.1	*Theropithecus gelada*	Mammal	XP_017814020.2	*Papio anubis*	Mammal
XP_011714843.1	*Macaca nemestrina*	Mammal	XP_011837025.1	*Mandrillus leucophaeus*	Mammal
XP_014996434.1	*Macaca mulatta*	Mammal	XP_011935444.1	*Cercocebus atys*	Mammal
XP_005557868.1	*Macaca fascicularis*	Mammal	XP_011761253.1	*Macaca nemestrina*	Mammal
XP_017387838.1	*Cebus imitator*	Mammal	XP_007978101.2	*Chlorocebus sabaeus*	Mammal
XP_012293527.1	*Aotus nancymaae*	Mammal	XP_005544418.1	*Macaca fascicularis*	Mammal
XP_023051577.1	*Piliocolobus tephrosceles*	Mammal	XP_017390136.1	*Cebus imitator*	Mammal
XP_010336924.1	*Saimiri boliviensis boliviensis*	Mammal	XP_032156810.1	*Sapajus apella*	Mammal
XP_032103933.1	*Sapajus apella*	Mammal	XP_039086462.1	*Hyaena hyaena*	Mammal

XP_003732249.3	*Callithrix jacchus*	Mammal	XP_003934868.1	*Saimiri boliviensis boliviensis*	Mammal
XP_004420239.1	*Ceratotherium simum simum*	Mammal	XP_042808605.1	*Panthera leo*	Mammal
XP_008689814.1	*Ursus maritimus*	Mammal	XP_040321713.1	*Puma yagouaroundi*	Mammal
XP_029073506.1	*Monodon monoceros*	Mammal	XP_042852848.1	*Panthera tigris*	Mammal
XP_036137777.1	*Molossus molossus*	Mammal	XP_022268687.1	*Canis lupus familiaris*	Mammal
XP_025850661.1	*Vulpes vulpes*	Mammal	XP_008564214.1	*Galeopterus variegatus*	Mammal
CAD7687878.1	*Nyctereutes procyonoides*	Mammal	XP_006189555.1	*Camelus ferus*	Mammal
XP_034870060.1	*Mirounga leonina*	Mammal	XP_002716171.1	*Oryctolagus cuniculus*	Mammal
XP_007172234.1	*Balaenoptera acutorostrata scammoni*	Mammal	XP_036991695.1	*Artibeus jamaicensis*	Mammal
XP_004744991.1	*Mustela putorius furo*	Mammal	XP_010992028.2	*Camelus dromedarius*	Mammal
XP_025288242.1	*Canis lupus dingo*	Mammal	XP_006196872.1	*Vicugna pacos*	Mammal
XP_021557701.1	*Neomonachus schauinslandi*	Mammal	XP_037684014.1	*Choloepus didactylus*	Mammal
XP_001502783.3	*Equus caballus*	Mammal	XP_032470576.1	*Phocoena sinus*	Mammal
XP_032281106.1	*Phoca vitulina*	Mammal	KAF6344155.1	*Rhinolophus ferrumequinum*	Mammal
XP_022444553.1	*Delphinapterus leucas*	Mammal	XP_030182683.1	*Lynx canadensis*	Mammal
XP_032693555.1	*Lontra canadensis*	Mammal	XP_032158516.1	*Mustela erminea*	Mammal
XP_035940195.1	*Halichoerus grypus*	Mammal	XP_014930621.1	*Acinonyx jubatus*	Mammal
XP_006731073.1	*Leptonychotes weddellii*	Mammal	XP_003989733.1	*Felis catus*	Mammal
XP_032192568.1	*Mustela erminea*	Mammal	XP_043430068.1	*Prionailurus bengalensis*	Mammal
XP_007461436.1	*Lipotes vexillifer*	Mammal	XP_025781566.1	*Puma concolor*	Mammal
XP_014683936.1	*Equus asinus*	Mammal	XP_019505662.1	*Hipposideros armiger*	Mammal
XP_025716622.1	*Callorhinus ursinus*	Mammal	XP_005895937.1	*Bos mutus*	Mammal
NP_001231228.1	*Sus scrofa*	Mammal	XP_006159497.1	*Tupaia chinensis*	Mammal
XP_004405521.1	*Odobenus rosmarus divergens*	Mammal	XP_004425827.1	*Ceratotherium simum simum*	Mammal
XP_027460775.2	*Zalophus californianus*	Mammal	XP_007459181.1	*Lipotes vexillifer*	Mammal
XP_040095998.1	*Oryx dammah*	Mammal	XP_025870268.1	*Vulpes vulpes*	Mammal
XP_011360644.1	*Pteropus vampyrus*	Mammal	XP_010828397.1	*Bison bison bison*	Mammal
XP_032483144.1	*Phocoena sinus*	Mammal	XP_019288059.1	*Panthera pardus*	Mammal
XP_002710148.1	*Oryctolagus cuniculus*	Mammal	XP_004592423.1	*Ochotona princeps*	Mammal
XP_027959315.1	*Eumetopias jubatus*	Mammal	XP_041623448.1	*Vulpes lagopus*	Mammal
XP_006923170.1	*Pteropus alecto*	Mammal	NP_001231209.1	*Sus scrofa*	Mammal
XP_019497692.1	*Hipposideros armiger*	Mammal	XP_036303654.1	*Pipistrellus kuhlii*	Mammal

XP_005890462.1	*Bos mutus*	Mammal	XP_032246641.1	*Phoca vitulina*	Mammal
XP_003404832.1	*Loxodonta africana*	Mammal	XP_027481384.1	*Zalophus californianus*	Mammal
XP_006042846.1	*Bubalus bubalis*	Mammal	XP_027969473.1	*Eumetopias jubatus*	Mammal
KAF6446151.1	*Rousettus aegyptiacus*	Mammal	NP_808534.1	*Mus musculus*	Mammal
NP_001192961.1	*Bos taurus*	Mammal	XP_021016344.1	*Mus caroli*	Mammal
XP_040300441.1	*Puma yagouaroundi*	Mammal	XP_031232783.1	*Mastomys coucha*	Mammal
XP_030725430.1	*Globicephala melas*	Mammal	XP_028615799.1	*Grammomys surdaster*	Mammal
XP_004282157.1	*Orcinus orca*	Mammal	XP_342942.5	*Rattus norvegicus*	Mammal
XP_026952872.1	*Lagenorhynchus obliquidens*	Mammal	XP_032744122.1	*Rattus rattus*	Mammal
XP_033709082.1	*Tursiops truncatus*	Mammal	XP_034359188.1	*Arvicanthis niloticus*	Mammal
XP_012617658.1	*Microcebus murinus*	Mammal	XP_005079712.1	*Mesocricetus auratus*	Mammal
XP_007087788.2	*Panthera tigris*	Mammal	XP_035294534.1	*Cricetulus griseus*	Mammal
XP_042106468.1	*Ovis aries*	Mammal	XP_041508774.1	*Microtus oregoni*	Mammal
XP_004475973.1	*Dasypus novemcinctus*	Mammal	XP_008835029.1	*Nannospalax galili*	Mammal
XP_014943117.1	*Acinonyx jubatus*	Mammal	XP_005353447.1	*Microtus ochrogaster*	Mammal
XP_003782369.1	*Otolemur garnettii*	Mammal	XP_021500052.1	*Meriones unguiculatus*	Mammal
XP_043444119.1	*Prionailurus bengalensis*	Mammal	XP_038190305.2	*Arvicola amphibius*	Mammal
XP_040128840.1	*Ictidomys tridecemlineatus*	Mammal	XP_028744060.1	*Peromyscus leucopus*	Mammal
EFB24281.1	*Ailuropoda melanoleuca*	Mammal	XP_036034511.1	*Onychomys torridus*	Mammal
XP_030174363.1	*Lynx canadensis*	Mammal	XP_004657367.1	*Jaculus jaculus*	Mammal
XP_025780691.1	*Puma concolor*	Mammal	XP_020014277.1	*Castor canadensis*	Mammal
VFV35163.1	*Lynx pardinus*	Mammal	XP_024410446.1	*Desmodus rotundus*	Mammal
XP_039098175.1	*Hyaena hyaena*	Mammal	KAF6107830.1	*Phyllostomus discolor*	Mammal
NP_001239416.1	*Mus musculus*	Mammal	XP_034503150.1	*Ailuropoda melanoleuca*	Mammal
XP_021036240.1	*Mus caroli*	Mammal	XP_004394766.1	*Odobenus rosmarus divergens*	Mammal
XP_021071551.1	*Mus pahari*	Mammal	XP_008693077.1	*Ursus maritimus*	Mammal
XP_034366447.1	*Arvicanthis niloticus*	Mammal	XP_026372328.1	*Ursus arctos horribilis*	Mammal
XP_006253634.1	*Rattus norvegicus*	Mammal	XP_034874899.1	*Mirounga leonina*	Mammal
XP_032740143.1	*Rattus rattus*	Mammal	XP_019836318.1	*Bos indicus*	Mammal
XP_031214101.1	*Mastomys coucha*	Mammal	XP_022407820.1	*Delphinapterus leucas*	Mammal
XP_006976830.1	*Peromyscus maniculatus bairdii*	Mammal	XP_004678705.1	*Condylura cristata*	Mammal
XP_028719682.1	*Peromyscus leucopus*	Mammal	XP_004478858.1	*Dasypus novemcinctus*	Mammal

XP_038191115.1	*Arvicola amphibius*	Mammal	XP_024622345.1	*Neophocaena asiaeorientalis asiaeorientalis*	Mammal
XP_041516240.1	*Microtus oregoni*	Mammal	XP_028936129.2	*Ornithorhynchus anatinus*	Mammal
XP_027263433.2	*Cricetulus griseus*	Mammal	XP_007491410.1	*Monodelphis domestica*	Mammal
XP_005078517.1	*Mesocricetus auratus*	Mammal	XP_020838635.1	*Phascolarctos cinereus*	Mammal
XP_012871450.1	*Dipodomys ordii*	Mammal	XP_038614576.1	*Tachyglossus aculeatus*	Mammal
XP_027796842.1	*Marmota flaviventris*	Mammal	XP_025952457.1	*Dromaius novaehollandiae*	Bird
XP_043321406.1	*Cervus canadensis*	Mammal	XP_009685997.1	*Struthio camelus australis*	Bird
XP_003980826.2	*Felis catus*	Mammal	XP_010212796.1	*Tinamus guttatus*	Bird
XP_028905845.1	*Ornithorhynchus anatinus*	Mammal	XP_023961582.2	*Chrysemys picta bellii*	Reptile
XP_007941994.1	*Orycteropus afer afer*	Mammal	XP_034609766.1	*Trachemys scripta elegans*	Reptile
XP_038595887.1	*Tachyglossus aculeatus*	Mammal	XP_039367103.1	*Mauremys reevesii*	Reptile
XP_025973941.1	*Dromaius novaehollandiae*	Bird	XP_030395237.1	*Gopherus evgoodei*	Reptile
XP_025919302.1	*Apteryx rowi*	Bird	XP_038233616.1	*Dermochelys coriacea*	Reptile
XP_013801334.1	*Apteryx mantelli mantelli*	Bird	KAG6932502.1	*Chelydra serpentina*	Reptile
NXE49475.1	*Casuarius casuarius*	Bird	TFK13269.1	*Platysternon megacephalum*	Reptile
XP_009679952.1	*Struthio camelus australis*	Bird	XP_027683016.3	*Chelonia mydas*	Reptile
XP_025901941.1	*Nothoprocta perdicaria*	Bird	XP_014379291.1	*Alligator sinensis*	Reptile
XP_010213286.1	*Tinamus guttatus*	Bird	XP_019345807.1	*Alligator mississippiensis*	Reptile
XP_009679952.1	*Struthio camelus australis*	Bird	XP_019403478.1	*Crocodylus porosus*	Reptile
XP_040459657.1	*Falco naumanni*	Bird	XP_015687548.1	*Protobothrops mucrosquamatus*	Reptile
KAG6941153.1	*Chelydra serpentina*	Reptile	XP_039190078.1	*Crotalus tigris*	Reptile
XP_034635897.1	*Trachemys scripta elegans*	Reptile	XP_025020920.1	*Python bivittatus*	Reptile
XP_023959442.1	*Chrysemys picta bellii*	Reptile	XP_015265723.1	*Gekko japonicus*	Reptile
XP_032639138.1	*Chelonoidis abingdonii*	Reptile	XP_020662583.1	*Pogona vitticeps*	Reptile
XP_030428818.1	*Gopherus evgoodei*	Reptile	XP_008122644.2	*Anolis carolinensis*	Reptile
XP_039341293.1	*Mauremys reevesii*	Reptile	XP_026558597.1	*Pseudonaja textilis*	Reptile
XP_014425482.1	*Pelodiscus sinensis*	Reptile	XP_034974637.1	*Zootoca vivipara*	Reptile

XP_043376637.1	*Chelonia mydas*	Reptile	KAG8143197.1	*Naja naja*	Reptile
XP_043346686.1	*Dermochelys coriacea*	Reptile	XP_032083635.1	*Thamnophis elegans*	Reptile
XP_006262243.1	*Alligator mississippiensis*	Reptile	XP_033014587.1	*Lacerta agilis*	Reptile
XP_025051820.1	*Alligator sinensis*	Reptile	XP_042314418.1	*Sceloporus undulatus*	Reptile
XP_019361807.1	*Gavialis gangeticus*	Reptile	KAG9279015.1	*Astyanax mexicanus*	Fish
XP_019390648.1	*Crocodylus porosus*	Reptile	XP_014033939.1	*Salmo salar*	Fish
XP_042310084.1	*Sceloporus undulatus*	Reptile	XP_038821756.1	*Salvelinus namaycush*	Fish
XP_015285132.1	*Gekko japonicus*	Reptile	XP_029588432.1	*Salmo trutta*	Fish
XP_020650120.1	*Pogona vitticeps*	Reptile	XP_024299172.2	*Oncorhynchus tshawytscha*	Fish
XP_003223983.1	*Anolis carolinensis*	Reptile	XP_019905824.2	*Esox lucius*	Fish
XP_032997734.1	*Lacerta agilis*	Reptile	XP_031650826.1	*Oncorhynchus kisutch*	Fish
>XP_028573915.1	*Podarcis muralis*	Reptile	XP_035617806.1	*Oncorhynchus keta*	Fish
XP_034961370.1	*Zootoca vivipara*	Reptile	XP_036809288.1	*Oncorhynchus mykiss*	Fish
XP_007431260.1	*Python bivittatus*	Reptile	XP_041758071.1	*Coregonus clupeaformis*	Fish
XP_039190780.1	*Crotalus tigris*	Reptile	XP_017570141.1	*Pygocentrus nattereri*	Fish
XP_029141623.1	*Protobothrops mucrosquamatus*	Reptile	XP_041096508.1	*Polyodon spathula*	Fish
XP_026551953.1	*Pseudonaja textilis*	Reptile	XP_036451899.1	*Colossoma macropomum*	Fish
XP_026540956.1	*Notechis scutatus*	Reptile	XP_015204558.1	*Lepisosteus oculatus*	Fish
XP_042604125.1	*Cyprinus carpio*	Fish	XP_041926155.1	*Alosa sapidissima*	Fish
XP_026051225.1	*Carassius auratus*	Fish	XP_014342686.1	*Latimeria chalumnae*	Fish
XP_043077188.1	*Puntigrus tetrazona*	Fish	XP_035385359.1	*Electrophorus electricus*	Fish
XP_010869366.1	*Esox lucius*	Fish	XP_031441058.2	*Clupea harengus*	Fish
XP_031664343.1	*Oncorhynchus kisutch*	Fish	XP_029514643.1	*Oncorhynchus nerka*	Fish
XP_014053738.1	*Salmo salar*	Fish	XP_034762146.1	*Acipenser ruthenus*	Fish
XP_029527704.1	*Oncorhynchus nerka*	Fish	XP_032900661.1	*Amblyraja radiata*	Fish
XP_014053737.1	*Salmo salar*	Fish			
XP_024250734.2	*Oncorhynchus tshawytscha*	Fish			
XP_041749357.1	*Coregonus clupeaformis*	Fish			
XP_041123027.1	*Polyodon spathula*	Fish			
XP_036845445.1	*Oncorhynchus mykiss*	Fish			
XP_029555370.1	*Salmo trutta*	Fish			
XP_006631912.1	*Lepisosteus oculatus*	Fish			
XP_041953876.1	*Alosa sapidissima*	Fish			
XP_035635474.1	*Oncorhynchus keta*	Fish			
XP_030645531.1	*Chanos chanos*	Fish			
XP_039598119.1	*Polypterus senegalus*	Fish			

XP_037399572.1	*Pygocentrus nattereri*	Fish			
XP_032885959.1	*Amblyraja radiata*	Fish			

Supplementary table 1: List of all the sequences from different species used in this study.

Catsper1				
	**Uniprot**	**TMHMM**	**DeepTMHMM**	**TMSEG**
TM1	448-469	443-465	447-468	450-468
TM2	479-500	486-508	480-500	482-502
TM3	509-531	Not detected	514-529	512-529
TM4	541-563	Not detected	541-551	549-561
TM5	582-604	583-605	583-601	580-601
		612-629		
TM6	646-671	644-666	647-667	649-671
**CatSper2**				
TM1	109-131	113-132	109-129	110-127
TM2	141-166	147-169	144-163	145-165
TM3	176-200	176-198	177-197	177-197
TM4	204-222	Not detected	205-215	210-222
TM5	240-262	241-263	243-263	241-262
		276-295		
TM6	315-341	315-337	318-338	319-339
**Catsper3**				
TM1	49-71	51-73	51-71	52-71
TM2	81-107	Not detected	83-100	85-104
TM3	109-131	114-131	116-131	115-134
TM4	144-160	Not detected	141-154	142-157
TM5	169-195	177-199	178-196	177-198
		209-226		
TM6	243-268	247-269	246-264	246-268
**Catsper4**				
TM1	91-112	93-112	93-111	93-111
TM2	123-149	127-149	125-145	127-146
TM3	154-177	156-173	158-178	157-176
TM4	181-199	Not detected	181-191	179-197
TM5	213-236	218-240	214-234	210-232
		247-264		
TM6	280-307	284-306	281-302	285-307

Supplementary table 2. Various software-based predictions of different transmembrane regions of human CatSper1, CatSper 2, CatSper 3, and CatSper 4. The transmembrane (TM) regions of all CatSperα were predicted using the TMHMM, Deep TMHMM, and TMSEG software. Some of these prediction-based TM regions differ by more than 5 amino acids than the experimentally characterized structure available in Uniport ID-Q8NEC5 for Catsper1, ID-Q96P56 for Catsper2, ID-Q86XQ3 for Catsper3, and ID-Q7RTX7 for Catsper4. Also, some of the transmembrane regions were not predicted at all by this software. Due to this variability, the human sequence from UniProt was used as the reference and all the transmembrane regions were identified across all the species for every analysis described in this paper.
